# Crossroads between Bacterial and Mammalian Glycosyltransferases

**DOI:** 10.3389/fimmu.2014.00492

**Published:** 2014-10-20

**Authors:** Inka Brockhausen

**Affiliations:** ^1^Department of Medicine, Queen’s University, Kingston, ON, Canada; ^2^Department of Biomedical and Molecular Sciences, Queen’s University, Kingston, ON, Canada

**Keywords:** glycosyltransferases, protein structure, specificities, glycoprotein epitopes, glycan mimics

## Abstract

Bacterial glycosyltransferases (GT) often synthesize the same glycan linkages as mammalian GT; yet, they usually have very little sequence identity. Nevertheless, enzymatic properties, folding, substrate specificities, and catalytic mechanisms of these enzyme proteins may have significant similarity. Thus, bacterial GT can be utilized for the enzymatic synthesis of both bacterial and mammalian types of complex glycan structures. A comparison is made here between mammalian and bacterial enzymes that synthesize epitopes found in mammalian glycoproteins, and those found in the O antigens of Gram-negative bacteria. These epitopes include Thomsen–Friedenreich (TF or T) antigen, blood group O, A, and B, type 1 and 2 chains, Lewis antigens, sialylated and fucosylated structures, and polysialic acids. Many different approaches can be taken to investigate the substrate binding and catalytic mechanisms of GT, including crystal structure analyses, mutations, comparison of amino acid sequences, NMR, and mass spectrometry. Knowledge of the protein structures and functions helps to design GT for specific glycan synthesis and to develop inhibitors. The goals are to develop new strategies to reduce bacterial virulence and to synthesize vaccines and other biologically active glycan structures.

## Introduction

Glycans play important roles in most biological processes in health and disease. Bacteria and human beings have a close relationship in the intestine, which can be symbiotic or pathogenic. Bacteria often produce human-like glycan structures with bacteria-specific glycosyltransferases (GT)s that have given them a selective advantage for adhesion, colonization, and survival. Knowledge of these enzymes can help us understand human counterparts of GTs, and provide a convenient technology to synthesize both bacterial and human glycans. Bacterial GTs can be easily expressed and stored; they are more soluble and often remarkably active and stable.

Currently, GTs from many different organisms have been classified into 96 GT families in the Carbohydrate-active Enzymes (CAZy) classification system (http://www.cazy.org), based on their sequence similarity derived from GenBank (ftp://ftp.ncbi.nih.gov/genbank/ or EMBL or DDBJ) (1, 2). Very few of the bacterial GTs have been biochemically and functionally characterized, thus proposed enzymes are assigned based on similarity searches. The CAZy database also contains genetic, structural, mechanistic, and functional information of known GTs. The former *Escherichia coli* (EC) nomenclature for GTs as well as the currently accepted nomenclature and alternative names for GTs are included. A number of databases provide sequence analyses of GTs (e.g., NCBI BLAST, PFAM, INTERPRO, DBCAN, Swiss-Prot – ExPASy).

For searches of glycan structures, a number of databases are useful (3). For example, GLYCOSuiteDB contains information on N- and O-linked glycans and glycoproteins and Glycobase on N- and O-Glycan structures. For glycomics analyses by mass spectrometry (MS), GlycoMaster DB at http://www-novo.cs.uwaterloo.ca:8080/GlycoMasterDB is helpful (4). The current *E. coli* O-antigen database (ECODAB) contains known O antigen structures of *E. coli*, the analytic data available, and has links to genes involved in O antigen synthesis from the O antigen gene cluster (5). Many of the *E. coli* antigens can be found in other bacterial strains. Finally, the Consortium for Functional Glycomics (http://www.functionalglycomics.org/) provides a large database for glycan functions.

Because of wide-spread development of antibiotic resistance, we need new anti-bacterial strategies, and bacterial GTs are virulence factors that could be targeted. The understanding of GTs can help in the production of vaccines to protect against bacterial infections, cancer, and for application in inflammation and autoimmune disease. In this review, we will compare mammalian and bacterial GTs that show remarkable similarity of action, protein folding, or mechanisms, in spite of surprisingly large differences in amino acid sequences.

## Mammalian Glycoproteins and Bacterial Glycans

Mammalian glycoproteins are involved in virtually all cellular activities; they serve as ligands for antibodies or lectins, or as receptors involved in signaling, cellular interactions, cell growth, differentiation, and cell death (6–11). Glycans are important in the inflammatory response, the innate and adaptive immune system, and cancer metastasis, as well as microbial colonization and infections. Glycoproteins have many functional epitopes attached to either N-glycans or O-glycans, and the amounts of many of these epitopes can be altered in disease, for example, in cancer. Although there is remarkable diversity in glycan structures in mammals, and hundreds of different chains can be found in glycoproteins, only six sugar residues (Man, GlcNAc, GalNAc, Gal, sialic acid, Fuc) are forming the extended and branched varieties of glycans with few modifications such as O-acetylation and sulfation. N- and O-glycans can affect the chemical and physical properties and the conformations of proteins and the accessibility of peptide epitopes.

Bacteria display an astounding variety of unusual sugars and sugar linkages as well as modifications of sugars that are foreign to human beings and, therefore, can trigger immune responses. However, a number of specific bacterial glycans are mimics of mammalian glycoprotein epitopes (Table 1). Partial structures of O-antigenic polysaccharides of Gram-negative bacteria (ECODAB) often mimic human glycans and may help bacteria to evade the immune system and promote colonization. The mimicry may prevent the production of effective vaccines to protect against bacterial infections, which requires new considerations of anti-bacterial strategies. About half of the *EC* strains have some form of mammalian epitope within their O antigens. This includes Galβ1-3GlcNAcβ-, and Galβ1-4GlcNAcβ-linkages, which are part of the glycan backbone structures (type 1 and type 2, respectively) in mammalian glycoproteins. In bacteria, those are internal structures within the O antigen repeating unit. The cancer-associated Thomsen–Friedenreich (TF or T antigen, O-glycan core 1) is common in glycoproteins and also found in several O antigens of *E. coli*. Blood group O, A, and B, sialylated glycans, and polysialic acid are mimics found in a number of bacterial strains. The fact that bacteria are able to synthesize these human-like structures suggests that they have the appropriate biosynthetic enzymes (Table 2), although this would be difficult to anticipate from the inspection of the amino acid sequences of their GTs. Biochemical characterization of bacterial enzymes and structure/function studies are important prerequisites to utilize these enzymes in chemoenzymatic synthesis of mammalian glycoprotein epitope structures.

**Table 1:** **Glycan mimics: examples of mammalian glycoprotein epitopes also found in the lipopolysaccharides (LPS) or lipooligosaccharides of Gram-negative bacteria**.

Glycoprotein epitope	Structure	Bacteria
T antigen	Galβ1–3GalNAcα-	*EC O104, EC O5, EC 0127*
Sialyl-T antigen	Sialylα2–3Galβ1–3GalNAcα-	*EC O104*
Type 1 chain	Galβ1–3GlcNAc-	*EC O7, EC O55*
Type 2 chain	Galβ1–4GlcNAc-	*EC O91*
Lewis a	Fucα1–4[Galβ1–3]GlcNAcβ-	*Hp*
Lewis b	Fucα1–4[Fucα1–2Galβ1–3]GlcNAcβ-	*Hp*
Lewis x	Galβ1–4[Fucα1–3]GlcNAcβ-	*Hp*
Lewis y	Fucα1–2Galβ1–4[Fucα1–3]GlcNAcβ-	*Hp*
H antigen	Fucα1–2Galβ-	*EC O86*
Blood group A	GalNAcα1–3[Fucα1–2]Galβ-	*Bo*
Blood group B	Galα1–3[Fucα1–2]Galβ-	*EC O86*
Linear B	Galα1–3Galβ-	*Nm*
P blood group	Galα1–4Galβ-	*Cj, Nm*
Polysialic acids, PSA	[Sialylα2–8]n	*EC, Nm*
Sialylα2-3	Sialylα2–3Galβ-	*Cj, EC O104*
Sialylα2-6	Sialylα2–6Galβ-	*Psp*
Fucα1-6	Fucα1–6GlcNAcβ-	*Rsp*

**Bo, Bacteroides ovatus*; *Cj, Campylobacter jejuni*; *EC, Escherichia coli*; *Hp, Helicobacter pylori*; *Nm, Neisseria meningitides*; *Psp, Photobacterium* sp.; *Rsp, Rhizobium* sp*.

**Table 2:** **Comparison of characterized mammalian and bacterial enzymes that catalyze a similar reaction**.

Human enzyme (Accession No.)	Acceptor	**Bacterial enzyme** *(Species)* **(Accession No.)**	Acceptor	Identity
Core 1 β3-Gal-transferase, C1GALT1, T synthase **(NP_064541, Q9NS00)**	GalNAcα-	WbwC (*EC*O104) **(AAK64375, Q93NP5)**	GalNAcα-PP-lipid	**10.5**
		WbiP (*EC*O127) **(YP_002329684, B7UT68)**	GalNAcα-R	**10.6**
		CgtB (*Cj*) **(ABL75151**, **A2I3T3)**	GalNAcβ-R	**8.4**
β4-Gal-transferase 1, B4GALT1 **(P15291)**	GlcNAcβ-	LgtB (*Nm*) **(EJU76624, J8YER7)**	GlcNAcβ-R	**9.3**
		WfeD (*Sb*) **(ACD37055, B5L3X1)**	GlcNAcα-PP-R	**8.6**
β3-Gal-transferase 5, B3GALT5 **(NP_149361**, **Q9Y2C3)**	GlcNAcβ-	WbgO (*EC*O55) **(ADD57106, D3QY14)**	GlcNAcβ-R	**11.4**
		WbbD (*EC*O7) **(AAC27537, Q03084)**	GlcNAcα-PP-lipid	**13**
β3-GlcNAc-transferase 2, B3GNT2 **(AAD09764, Q9NY97)**	Galβ-	LgtA (*Nm*) **(AAL12840, Q93EK6)**	lactose	**11.6**
α3-Gal-transferase, ABO transferase GTB **(AAD26574, Q9UQ64)**	Fucα2Galβ-	WbnI (*EC*O86) **(AAV80756, Q5JBG6)**	Fucα2Galβ-R	**20.6**
α4-Gal-transferase, A4GALT **(AAO39150, Q540I6)**	Galβ-	CgtD (*Cj)* **(AAM90647, Q8KWQ9)**	Galβ-R	**11.6**
		LgtC (*Nm*) **(AAL12839, Q93EK7)**	Galβ-R	**10.7**
α3-GalNAc-transferase, ABO transferase GTA (**V5ZDN8, P16442)**	Fucα2Galβ-	BoGT56a (*Bo*) **(4AYL_A, A7LVT2)**	Fucα2Galβ-R	**21.1**
		GTA-like (*Hm*) **(CBG40459, D3UIY4)**	Fucα2Galβ-R	**19.7**
α3-Sialyl-transferase ST3GAL1 **(NP_775479, Q11201)**	Galβ3GalNAc-	NST (*Nm*) (**C6S6J1)**	Lactoseβ-benzyl	**13.2**
		WbwA (*EC*O104) **(AAK64371, Q93NP9)**	Galβ3GalNAcα-PP-lipid	**13.6**
α3-Sialyl-transferase, ST3GAL4 **(Q11206)**	Galβ3-	CstI (*Cj*) **(ADA70348, D2KQ02)**	Galβ3-R	**10.2**
α6-Sialyl-transferase, ST6GAL1 **(P15907)**	Galβ3-	Pm0188 (*Pm*) **(Q9CP67)**	Galβ-R	**14.6**
		α6Sialyltransferase (*Psp*) **(A8QYL1)**	Galβ-R	**14.6**
Poly α8-sialyl-transferase 2, ST8SIA2 **(AAH10645, Q92186)**	sialyl-Gal-	PST (*Nm*) **(AAS90326, Q6PUE5)**	GD1 ganglioside	**9.8**
		PST (*EC*) **(B1LEB7)**	Sialyl-R	**5.4**
α2-Fuc-transferase, FUT2 **(CAQ81982, A8K2L2)**	Galβ-	WbwK (*EC*O86) (**AAO37719, Q5JBG3)**	Galβ3GalNAc-	**11.9**
		WbsJ (*EC*O128) **(AAO37698, Q6XQ53)**	Galβ4-R	**15.8**
		FutC (*Hp*) **(ABO61750, A4L7J1)**	Galβ-	**17.5**
α3-Fuc-transferase, FUT4 **(NP_002024, P22083)**	Galβ4GlcNAcβ-	FutA (*Hp)* (**YP_003057735, C7BXF2**)	Galβ4GlcNAcβ-R	**10**
		FutB (*Hp)* **(YP_003057467, C7BZU7)**	Galβ4GlcNAcβ-R	**10.2**
		α3FUT (*Hh)* **(Q7VFA1)**	(Sialyl-)Galβ4GlcNAcβ-R	**10.4**
α3/4-Fuc-transferase, FUT3 **(P21217)**	Galβ4/3GlcNAcβ-	FucTa (*Hp)* **(AAF35291, Q9L8S4)**	Galβ4/3GlcNAcβ-R	**14.5**
α6-Fuc-transferase, FUT8 **(AAI42959, A5PLL2)**	N-glycan core	NodZ (*Rsp*) **(G9ACH1)**	Chitobiose	**7.6**

*The donor substrates of mammalian and bacterial enzymes that synthesize the same linkage are the same but the preferred acceptor substrate may differ. The bacterial enzyme can mimic the mammalian enzyme and can be used to synthesize mammalian glycan structures but the amino acid sequences have little sequence identities. The percentage of amino acid identity is much higher among eukaryotic GT families and between GTs with similar action and using similar acceptors in bacteria. The accession numbers in bold (Accession No.) are from Pubmed and/or UniProt database. *Bo, Bacteroides ovatus*; *Cj, Campylobacter jejuni*; *EC, E. coli*; *Hh, Helicobacter hepaticus*; *Hm, Helicobacter mustelae*; *Hp, Helicobacter pylori*; *Nm, Neisseria meningitides*; *Pm, Pasteurella multocida*; *Psp, Photobacterium* sp.; *Rsp, Rhizobium* sp.; *Sb, Shigella boydii*; PP-, diphosphate-*.

### Role of O antigens

The LPS of Gram-negative bacteria are essential structures of the outer membrane. LPS binds to the LPS-binding protein, requiring the CD14/TLR4/MD2 receptor complex, which elicits a strong response during infections, through TLR4 signaling. LPS consist of a lipid A base (endotoxin), which carries a relatively invariable inner oligosaccharide core, strain-specific outer core oligosaccharides, and the serotype-specific outer O antigen polysaccharide. O antigens are polysaccharides composed of up to 50 repeating units of oligosaccharides with one (homopolymeric) to 10 sugars (heteropolymeric) that play a role in bacterial adhesion and colonization, affect pathogenicity and survival, and can be bacteriophage receptors. The enormous structural variability of O antigens is mediated by many specific GTs and other enzymes that modify O antigens, thus increasing structural diversity, e.g., by adding phosphate, acetyl groups, or branching sugar residues. The LPS molecules are necessary for stabilization of the outer membrane and form a barrier against penetration of toxins. In particular, the O antigens serve to evade complement; they protect against phagocytosis and give the bacteria a strain-specific and diversity-selective advantage. The molecular mimicry found in a large proportion of bacteria adds to their ability to prevent recognition by the host immune system and thus promotes virulence.

A number of bacteria do not have an extended O-antigenic polysaccharide but instead have a short lipooligosaccharide that may have structural identity with human glycoproteins or glycolipids and may lead to pathological conditions. The close relationship between bacteria and human beings is also apparent in the abundance of bacterial lectins that bind to mammalian glycoproteins and thus promote adhesion to mammalian tissues.

### N-Glycosylation of mammalian and bacterial glycoproteins

**Figure 1:**
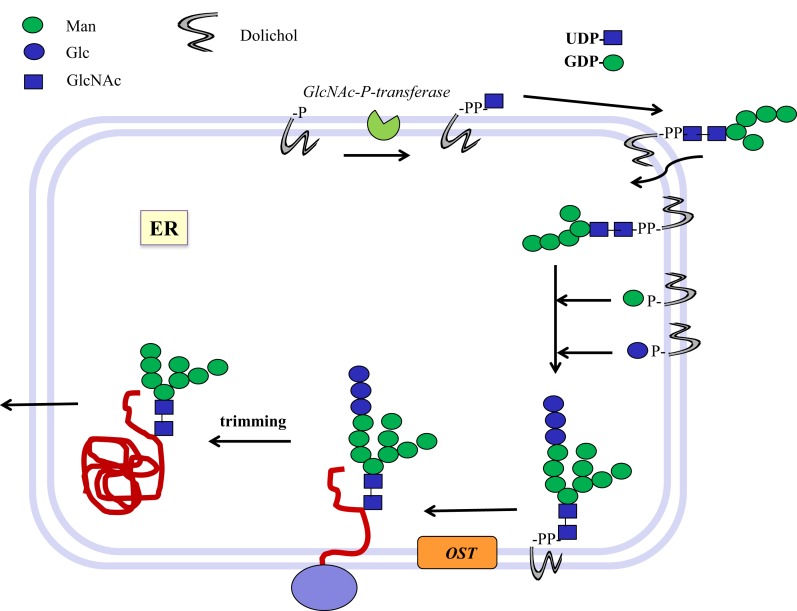
**Biosynthesis of N-glycosylated glycoproteins in eukaryotes**. N-glycosylation is initiated at the endoplasmic reticulum (ER) membrane using nucleotide sugar donor substrates and a membrane-bound acceptor phospholipid with multiple isoprenyl units (dolichol-phosphate, P-Dol). The first sugar (GlcNAc) is transferred as GlcNAc-phosphate from UDP-GlcNAc by GlcNAc-P-transferase, resulting in GlcNAc-diphosphate-dolichol (GlcNAc-PP-Dol). This step can be inhibited by the UDP-GlcNAc analog tunicamycin. On the outside face of the ER membrane, another GlcNAc is added to form chitobiose, followed by five Man residues to form a heptasaccharide (Man_5_GlcNAc_2_)-PP-Dol. This heptasaccharide is flipped to the inside of the ER where the chain grows by transfer of sugars from membrane-bound Man-P-Dol and Glc-P-Dol. The completed saccharide Glc_3_Man_9_GlcNAc_2_ is then transferred by an oligosaccharyltransferase complex (OST) to the Asn residue in an Asn-x-Ser/Thr sequon of nascent proteins. After trimming of sugar residues in the ER by removal of Glc and Man residues to the Man_8_GlcNAc_2_ structure, glycoproteins are exported to the Golgi where further trimming occurs by mannosidases. Many N-glycan chains are processed to the complex type by the addition of GlcNAc residues by GlcNAc-transferases I to V (MGAT1 to 5). Chains grow further by the addition of Gal-GlcNAc sequences and termination by sialyl-, Fuc-, Gal-, GlcNAc-, and GalNAc-transferases, which are all highly specific for both the donor and the acceptor substrates and with few exceptions form only one type of linkage between sugars. This creates a multitude of hundreds of different structures and epitopes with many possible functions, depending on the final destination of the glycoprotein, e.g., in the cell membrane or in secretions. Glycoprotein biosynthesis is regulated at many different levels, e.g., by the synthesis and delivery of nucleotide sugar substrates, the expression, activities and localization of glycosyltransferases and trimming hydrolases, the competition of enzymes for common substrates, levels of metal ion activating factors, localization of enzymes involved, and rate of transport of glycoproteins.

In eukaryotic cells, N-glycans are assembled first on a dolichol-phosphate (P-Dol) intermediate on the cytoplasmic side of the endoplasmic reticulum (ER) membrane (7, 12) (Figure 1). GlcNAc-phosphate is transferred by GlcNAc-phosphotransferase in a reversible reaction, inhibited by tunicamycin, to P-Dol, followed by the transfer of another GlcNAc residue to form chitobiose. This is followed by five Man residues, all transferred from nucleotide sugar donor substrates to form the common N-glycan core structure Man_5_-chitobiose linked to PP-Dol. This heptasaccharide is flipped across the membrane and further addition of sugars occurs on the inside of the ER lumen through transfer from Man-P-Dol and Glc-P-Dol donor substrates. After completion of the lipid-linked N-glycan, it is transferred *en bloc* to the Asn residue(s) of Asn-X-Ser/Thr sequons in a glycoprotein by the oligosaccharyltransferase complex (OST), and Glc and Man residues are selectively cleaved by glycosidases. After transfer to the Golgi, further removal of Man residues occurs, and GlcNAc-transferase I (GnT I, MGAT1) adds the first of the N-glycan antennae in β1–2 linkage to the Manα1–3 arm of the core. This can then be followed by several steps that depend on the presence of this first GlcNAc antenna and the expression of processing enzymes, which remove two Man residues from the Manα1–6 arm, add Fucα1–6 to the inner chitobiose GlcNAc, and add further antennae to form complex type N-glycans. N-glycans can be extended by repeating Gal-GlcNAc residues to form type 1 or type 2 chains (Table 1); they may be branched by GlcNAcβ1–6 linkages and may be decorated with specific functional epitopes and blood group determinants (7). This results in hundreds of different N-glycan structures, depending on the glycosylation potential of the cell.

Not all N-glycosylation sites carry N-glycans, and there are differences in chain processing between different glycosylation sites of the same protein. The peptide has been shown to interact with the glycan chains, and this controls the conformations of the glycan and the peptide and leads to site-specific glycosylation. Many sequentially acting and competing GTs assemble glycoproteins in a cell type-specific pattern. Most of the GTs involved exist as families of enzymes (Table 3). Several of these have been shown to be localized in specific Golgi compartments according to their action within the complex pathways.

**Table 3:** **Examples of glycosyltransferase families (CAZy)**.

GT family	Mechanism	Fold	Glycosyltransferases	Known structures
2	I	GT-A	WbbD, WbwC, WbiP, CgtB, WbgO, WfaP, WfgD, LgtA	7
6	R	GT-A	GTA, GTB, A3GALT, WbnI, BoGT56a, GTA-like (*Hm*)	4
7	I	GT-A	B4GALT	4
8	R	GT-A	LgtC	5
10	I	GT-B	α3/4FUT, FutA, FutB, FucTa, FUT3, FUT4-7, 9-11	1
11	I	GT-B	α2FUT, α3FUT (*Hh*), WbwK, Wbsj, FutC	–
13	I	GT-A	GnT I, MGAT1	1
14	I	GT-A	C2GnT1, IGnT, GCNT2	1
16	I	?	MGAT2	–
17	I	?	MGAT3	–
18	I	?	MGAT5	–
23	I	GT-B	α6FUT8, NodZ	2
25	I	?	LgtB	–
26	I	?	WfeD	–
27	R	GT-A	GALNT	3
29	I	GT-A	Animal sialyl-T, ST3GAL, ST6GAL, ST6GALNAC, ST8SIA	3
31	I	GT-A	C1GALT, B3GALT5	1
32	R	?	CgtD, Lgt5, A4GALT	–
38	I	?	Bacterial PST	–
42	I	GT-A	Bacterial Sialyl-T, CstI, CstII	2
52	I	GT-B	Bacterial Sialyl-T, NST, WbwA	1
54	I	?	MGAT4	–
80	I	GT-B	Bacterial Sialyl-T, Pm0188, α6Sialyl-T (*Psp*), PmST1, multifunctional	4

*Fold, expected overall fold; *Hh, Helicobacter hepaticus*; *Hm, Helicobacter mustelae*; I, Inverting mechanism; *Psp, Photobacterium* sp.; R, Retaining mechanism; -T, -transferase*.

In the mammalian biosynthetic pathways, the sequence of sugar additions is controlled by the gene expression, the relative activities of competing enzymes, the enzyme localizations, levels of substrates and cofactors, and the distinct substrate specificities of GTs. These types of controls still need to be investigated for glycosylation reactions in bacteria.

Bacteria such as *Campylobacter jejuni* (*Cj*) also have N-glycosylated proteins (13). An oligosaccharide is first assembled on undecaprenol-phosphate (P-Und), an analog of P-Dol, in the cytoplasmic compartment. The sugar-PP-Und is then flipped to the periplasmic space where the glycan chain is transferred *en bloc* to protein by oligosaccharyltransferases. These GTs have a broad specificity toward their donor substrates but also require a sequon, Asp/Glu–x–Asn–y-Ser/Thr, where x and y cannot be Pro, in the protein acceptor, that bears close resemblance to the mammalian N-glycosylation sequon (Figure 1).

### Protein O-glycosylation

O-glycans of glycoproteins and mucins are assembled in mammals without a lipid intermediate and without removal of sugar residues by glycosidases (14, 15). The first sugar is always GalNAcα-linked to Ser or Thr (the cancer-associated Tn antigen). All sugars are transferred from nucleotide sugars in the Golgi, resulting in extended and branched O-glycans with hundreds of different structures. The most common structure is Galβ1-3GalNAc, core 1, the T antigen, which is normally masked by the addition of other residues but exposed in many cancer cells. In a number of cells, core 1 is branched by core 2 β6-GlcNAc-transferase (C2GnT) or extended in a fashion that is similar to the synthesis of complex N-glycans.

GalNAc is transferred from UDP-GalNAc by up to 20 polypeptide GalNAc-transferases (GALNTs) (14–16). All GALNTs are classified in the GT27 family with a GT-A fold (Table 3). They have a catalytic domain linked to a lectin (ricin-like) domain at the C terminus. This lectin domain has three subdomains and may play an important role in binding products or substrates containing GalNAc residues. A crystal structure of mouse GALNT1 with Mn^2+^ supported the importance of a DxH motif and the role of Asp209, His211, and His344 (17) (Table 4). The conformations of human GALNT2 (18) crystallized with UDP and with or without an acceptor peptide showed a loop formed over UDP. It appeared that the acceptor peptide connected the otherwise separate catalytic and lectin domains. Kinetic analyses showed that the presence of GalNAc in the acceptor was beneficial for activity. Human GALNT10 was crystallized complexed with UDP, GalNAc, and Mn^2+^ (19). GalNAc-peptides appear to bind to the second beta-subdomain of the lectin domain. Binding of the donor induces a conformation change that opens the acceptor-binding site. These three crystallized ppGalNAcTs are similar in overall structure and mechanism.

**Table 4:** **Mammalian and bacterial glycosyltransferases with known crystal structure relevant to glycoprotein determinants or their mimics**.

Enzyme name		Species	Donor	Acceptor	GT	Fold	DxD	Reference
**INVERTING**								
GnT I, MGAT1		Rabbit	UDP-GlcNAc	Manα3-R	13	GT-A	DxD	(19)
C2GnT, GCNT1		Mouse	UDP-GlcNAc	Galβ3GalNAc-R	14	GT-A	E320	(20, 21)
β4-Gal-T1, B4GALT1		Human	UDP-Gal	GlcNAcβ-R	7	GT-A	DxD	(22)
β4-Gal-T1, B4GALT1		Bovine	UDP-Gal	GlcNAcβ-R	7	GT-A	DxD	(23)
β4-Gal-T7, B4GALT7		Human	UDP-Gal	GlcNAcβ-R	7	GT-A	DxD	(24)
α3-FUT		*Hp*	GDP-Fuc	Galβ4GlcNAc	10	GT-B	E95, E249	(25)
α6-FUT8		Human	GDP-Fuc	N-glycan core Gn	23	GT-B	D453	(26)
NodZ		Rsp	GDP-Fuc	chitobiose	23	GT-B		(27)
ST3GAL1		Pig	CMP-SA	Galβ3GalNAc-R	29	GT-A	H319	(28)
α3-Sialyl-T		*Pp*	CMP-SA	Gal-R	80	GT-B	H317	(29)
α3-Sialyl-T	CstI	Cj	CMP-SA	Galβ-R	42	GT-A	H202	(30)
α3-Sialyl-T	NST	Nm	CMP-SA	Lactose-Bn	52	GT-A	H280	(31)
ST6GAL1		Rat	CMP-SA	Galβ4GlcNAc	29	GT-A	H367	(32)
ST6GAL1		Human	CMP-SA	Galβ4GlcNAc	29	GT-A	H370	(33)
α6-Sialyl-T	PM0188	*Pm*	CMP-SA	Lactose, Gal, GalNAc, SA	80	GT-B	D141	(34)
α6-Sialyl-T	pst6-224	*Psp*	CMP-SA	Lactose	80	GT-B		(35)
α3/8-Sialyl-T	CstII	*Cj*	CMP-SA	Galβ-R	42	GT-A	H188	(36)
α3-Sialyl-T multi	PmST1	*Pm*	CMP-SA	Lactose, Gal, GalNAc, sialic acid	80	GT-B	D141	(37)
**RETAINING**								
GALNT1		Mouse	UDP-GalNAc	Thr/Ser	27	GT-A-ricin		(17)
GALNT2		Human	UDP-GalNAc	Thr/Ser	27	GT-A-ricin		(18)
GALNT10		Human	UDP-GalNAc	Thr/Ser	27	GT-A-ricin		(38)
ABO transferase	GTB	Human	UDP-Gal	H antigen	6	GT-A	E303	(39)
							H347	(40)
α3-Gal-T	Linear B	Bovine	UDP-Gal	Galβ4GlcNAc	6	GT-A	E317, H271	(41, 42)
	Gal-T							
ABO transferase	GTA	Human	UDP-GalNAc	H antigen	6	GT-A	E303	(39, 43)
							H347	
α3-GalNAc-T	BoGT6a	*Bo*	UDP-GalNAc	Fuc-lactose	6	GT-A	NxN	(44)
α4-Gal-T	LgtC	*Nm*	UDP-Gal	Lactose, oligosac	8	GT-B	4DxD, H188	(45)

*Enzymes are listed that have been characterized and crystallized and shown to be involved in the synthesis of glycoproteins or their glycan mimics in bacteria. *Bo, Bacteroides ovatus*; *Bs, bacillus subtilis*; *Cj, Campylobacter jejuni*; DxD, presence of a DxD motif or its analog or proposed catalytic residue; GT, Glycosyltransferase family (CAZy); *Hp, Helicobacter pylori*; multi, multifunctional enzyme; Inverting: enzymes invert the anomeric configuration of the sugar of the donor substrate to form enzyme product; N-glycan core Gn, reducing GlcNAc residue of the N-glycan core structure; *Nm, Neisseria meningitides*; *Pm, Pasteurella multocida*; *Pp, Photobacterium phosphoreum*; *Psp, Photobacterium* sp.; *Rsp, Rhizobium* sp.; Retaining: enzymes retain the anomeric configuration of the sugar of the donor substrate in the enzyme product; SA, sialic acid; -T, -transferase*.

An equivalent GALNT that transfers GalNAc to protein has not been identified in bacteria, although bacteria are known to O-glycosylate Ser/Thr residues of proteins with various sugar residues. In contrast to mammalian O-glycosylation, bacteria transfer a pre-assembled oligosaccharide to Ser/Thr. Bacterial protein OGTs have no sequence homology to GALNT and their action is reminiscent to that of OST in the N-glycosylation pathway. In several bacteria, for example in *Campylobacter* and *Neissseria*, an oligosaccharide or monosaccharide is first pre-assembled on PP-lipid in the cytoplasmic compartment, flipped to the periplasm and then transferred *en bloc* to Ser/Thr residues of proteins. These enzymes have a relaxed oligosaccharide donor specificity (46). Oligosaccharyltransferase PglL (which has not yet been assigned to a GT family) from *Neisseria meningitides* (*Nm*) can transfer many different glycans from sugar-PP-Und or sugar-PP-lipid (including sugar-PP-Dol) donor substrates to protein in the periplasmic space. UDP-*N*-diacetyl-bacillosamine was also a donor substrate *in vitro*, showing that even nucleotide sugars can be donors and a single sugar could be transferred to protein. Mutagenesis experiments showed that PglL from *Nm* requires His349 for activity and for interaction with the lipid-linked oligosaccharide (47).

### Biosynthesis of bacterial O antigens

**Figure 2:**
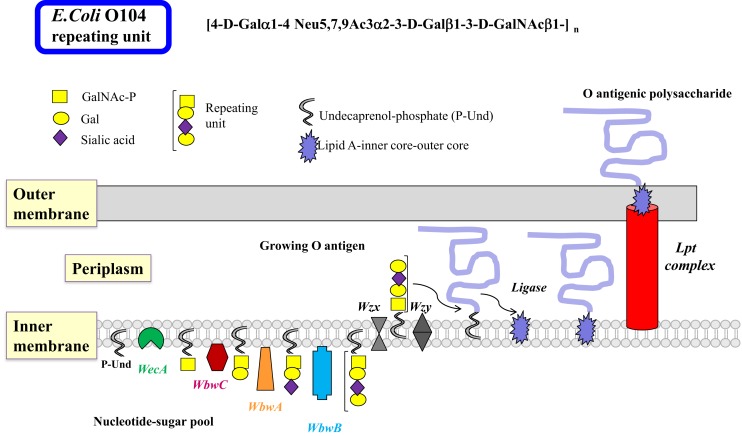
**Biosynthesis of lipopolysaccharides in Gram-negative bacteria by the polymerase-dependent pathway**. Many steps of the complex sequences and controls in the biosynthesis of LPS in Gram-negative bacteria are similar to those in mammalian glycoprotein biosynthesis. The inner membrane serves as the site of glycan biosynthesis, and the membrane-bound acceptor is undecaprenol-phosphate (P-Und) having 11 isoprenyl units, which is less than those found in eukaryotic Dol. Nucleotide sugars are synthesized in the cytosol and used for most glycosylation reactions. As in the N-glycan biosynthesis, the first sugar is transferred as sugar-phosphate by membrane-bound WecA to synthesize GalNAc/GlcNAc-PP-Und. This step can also be blocked by tunicamycin. It is possible that a 4-epimerase is involved. Subsequently, sugars are added individually to form the repeating unit of the O antigen. The glycosyltransferases that transfer sugars from nucleotide sugars usually have a high specificity for their donor and acceptor substrates and are associated with the membrane. After Wzx transports the repeating units to the periplasm, they are polymerized by Wzy by addition of repeating units to the reducing end of the growing polysaccharide linked to PP-Dol. The O antigen can be further processed and modified to form completed O antigens and the biosynthesis is usually terminated with Wzz. The O polysaccharide is then transferred to a sugar of the core oligosaccharide linked to lipid A by a ligase, forming the LPS, which is exported to the outer membrane by the Lpt complex. The O antigenic polysaccharide is then exposed to the environment on the outer membrane. Although many bacterial enzymes involved in LPS synthesis have been cloned, the individual steps of LPS synthesis are not well understood, mainly because of the major challenge to find the appropriate enzyme substrates and conditions to assay enzymes. The example shows the biosynthesis of the *E. coli* O104 antigen. The repeating unit tetrasaccharide contains the cancer-associated T antigen (Galβ1-3GalNAc), as well as the sialyl-T antigen (sialylα2-3Galβ1-3GalNAc). The WbwA sialyltransferase and the WbwB Gal-transferase remain to be characterized.

There are many similarities in the pathways and mechanisms by which bacterial O antigens and mammalian glycoproteins are synthesized. In Gram-negative bacteria, O antigens are synthesized by specific GTs at the cytosolic face of the inner membrane where the nucleotide sugar donor substrates are present, as well as the membrane-bound P-Und, an analog of the mammalian P-Dol, as the acceptor substrate for the transfer of the first sugar (48) (Figure 2). The first GT to act is always a sugar-phosphate transferase that produces the sugar-PP-Und substrate for subsequent transfer of monosaccharides by GTs. Most *E. coli* have GlcNAc or GalNAc at the reducing end of the repeating unit, thus sugar-phosphate transferase WecA and its orthologs are responsible for the first reaction, maintaining the α-anomeric configuration of GlcNAc. 4-Epimerases may also be involved in interconverting GlcNAc and GalNAc in the activated form (UDP-GlcNAc/UDP-GalNAc) or after the sugar transfer (49).

The common heteropolymeric O antigens are synthesized by sequential transfer of sugar units by donor- and acceptor-specific, membrane-associated GTs. The specificities of these bacterial GTs are distinct and comparable to eukaryotic GTs. A completed repeating unit is then translocated across the inner membrane to the periplasmic side by the multiple membrane-spanning flippase Wzx, a process resembling the transfer of Man_5_GlcNAc_2_–PP-Dol intermediate across the ER membrane. Polymerization involves the addition of repeating units to the reducing end of the growing chain by Wzy polymerase. This enzyme has 12 predicted transmembrane domains with the catalytic domain in the periplasm that has some specificity for the structure of the repeating unit. Wzy may invert the anomeric linkage of the first sugar in the polysaccharide since many repeating units have the GlcNAcβ-linkage in the O antigen. Many genes specifically involved in the synthesis of the O antigen are found in the O antigen gene cluster. The presence of the *wzy* gene suggests that the O antigen is synthesized by the Wzy-dependent pathway (Figure 2). A much less specific chain terminator Wzz then helps to restrict the number of repeating units assembled in the O antigen. This is followed by a ligase (polysaccharide transferase)-catalyzed transfer of the O antigen to a specific sugar of the outer core structure, synthesizing the complete LPS. This releases PP-Und, which is recycled to P-Und. LPS is then extruded to the outer membrane by the Lpt complex (50).

**Figure 3:**
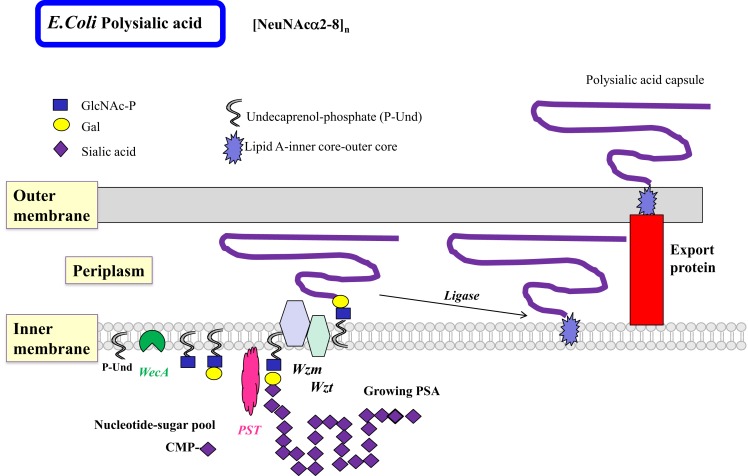
**Biosynthesis of polysialic acids in *E. coli* using the ABC transporter pathway**. The biosynthesis of homopolymeric O antigens (or capsules) is initiated at the cytosolic side of the inner membrane. Polysialic acids (PSA) of *E. coli* are proposed to be assembled in a processive fashion as shown, based on undecaprenol-phosphate. Membrane-associated polysialyltransferase (PST) transfers many units of sialic acid from CMP-sialic acid to the growing polysialic acid. The enzyme can act on a number of acceptor substrates to form repeated sialylα2–8 linkages. A termination reaction stops the growth of the long PSA chain. The PSA is then transported to the periplasmic space by the Wzm exporter, which is associated with the ATP-binding Wzt. Further processing occurs in the periplasm. The completed PSA is ligated to the core-lipid A and then translocated by export proteins to the outer membrane to serve as a highly charged and hydrophilic protective coat. Other homopolymeric O antigens such as poly-d-Mannose or poly-d-Rhamnose are processed in a similar fashion by the ABC transporter pathway.

The less common homopolymeric O antigens, such as the d-Rha polymers of *Pseudomonas aeruginosa* (*Pa*) and the d-Man polymers of *E. coli* O9, are synthesized by the transfer of monosaccharides from nucleotide sugars to R-GlcNAc-PP-Und in a processive fashion in the ABC transporter-dependent pathway (Figure 3) (51). Some of the processive GTs can have multiple catalytic domains, e.g., Man-transferase WbdA. The entire O antigen is assembled on the cytosolic side, and terminated by termination reactions, e.g., methylation. This is followed by translocation of the large O-antigen-PP-Und by the Wzm exporter and the ATP-binding Wzt to the periplasm where it is further processed. The presence of *wzm* and *wzt* genes in the O antigen gene cluster would suggest that this pathway is operative.

The events utilizing membrane-bound acceptor substrates in bacteria are similar to those of the early N-glycan synthesis in eukaryotes at the ER inner membrane (Figure 1). In both mammals and bacteria, isoenzymes are known that can synthesize the same linkage, often with slightly different substrate specificity. These isoenzymes are interesting models to study the catalytic sites and requirement for specific amino acids critical for catalysis and specificity.

## Characterization of Glycosyltransferases

Chemical synthesis has been used to produce natural-like or unnatural glycans but the stereochemistry and regio-selectivity is difficult to achieve. Nature has developed GTs, excellent tools to synthesize an amazing diversity of glycan structures with defined anomeric configurations and linkages. GT reactions do not require harsh conditions or protection of reactive groups. GTs have distinct specificities for their donor and acceptor substrates. More than 100,000 genes from various species are thought to encode GTs, and organisms have 1–2% of their genes dedicated to GTs (52).

In order to assess the requirements and characteristics of GT activities, specific and accurate enzyme assays have to be developed. Nucleotide sugar donor substrates for mammalian glycoprotein biosynthesis are usually commercially available but for bacterial enzymes may have to be chemically or enzymatically synthesized. It would be difficult to extract the natural donor and acceptor substrates from bacteria in the pure form. Therefore, syntheses for bacteria-specific donor substrate analogs have been developed, e.g., for UDP-QuiNAc (UDP-6-deoxy-GlcNAc) found in *E. coli* and *Pseudomonas aeruginosan* (*PA*) (53) or for GDP-d-Rha found in *PA* (54). Oligosaccharides linked to a synthetic aglycone group may be suitable acceptors for both, mammalian GTs and bacterial GTs. However, bacterial GTs that act early in the O antigen synthesis pathway seem to require sugar-diphosphate-lipids as acceptors, which are difficult to synthesize. We developed the natural acceptor analog GlcNAcα-diphosphate-lipid to mimic the product of the first sugar-phosphate addition (55), which was very active as an acceptor. In order to isolate the enzyme product from the assay mixture for quantification, a number of different chromatographic methods have been employed, including hydrophobic or anion exchange methods, HPLC, TLC, and capillary electrophoresis. Enzyme-coupled assays or lectin and antibody binding have also been used to determine activities. Methods to assay specific GTs are essential prerequisites to study their properties and optimal conditions, substrate specificities, and to develop inhibitors.

GTs can be classified based on similarities of their amino acid sequences, according to the sugar they transfer, and the stereochemistry of the reaction in the CAZy database. If at least 100 amino acids in two different stretches of the protein have significant similarity to other members of the same family but not to other families, GTs are assigned to a specific family with the same predicted fold, and being either inverting or retaining GT (Tables 3 and 4). However, not all known sequences fit into a GT family or are reclassified when the specific function of the GT has been established, and the number of families are growing. Sequence similarity of unknown proteins can be used to predict function and protein folding. However, the final proof of function has to be obtained by biochemical analysis of enzymes. Most GTs in bacteria have not been functionally characterized, and this area is both challenging and tedious, often because the appropriate donor and acceptor substrates have to be especially prepared.

Crystal structures for GTs from eukaryotic and prokaryotic sources have been helpful in delineating the catalytic actions of GTs. It is interesting that this large and important class of thousands of enzymes that bind to many different nucleotide sugars as well as to a very large variety of monosaccharides, oligosaccharides, glycopeptides, and glycolipids occurs in only two major fold types, GT-A and GT-B. GTs, thus, have a relatively conserved three-dimensional architecture within their catalytic sites and share mechanisms, resulting in an extremely large number of product structures with linear or branched glycans of mostly unknown functions.

The binding of substrates to GTs and the transfer reactions have been shown to involve conformational changes in the enzyme proteins. GT-A folded enzymes have two tightly associated α/βα Rossman nucleotide-binding-like domains with two α-helices surrounding an open twisted, central β-sheet. The donor and acceptor substrates bind in different domains. The GT-B folded enzymes have two β/α/β Rossman-like domains, which are less tightly associated with each other and have the active site in the cleft in between domains (56). Usually, the sugar donor substrate binds first. This induces a conformational change in the enzyme forming a lid over the nucleotide sugar, facilitating the binding of the acceptor substrate and catalysis in an ordered sequential, regio- and stereo-specific mechanism (57, 58). Internal disordered loops seem to be a common feature in mammalian and bacterial enzymes (40). Upon substrate binding, a disordered, short protein loop becomes ordered when donor substrate is bound. A change in orientation and conformation of the resulting ordered loop appears to facilitate binding of the second substrate and catalysis. Thus, the function of an ordered loop could be to allow catalysis, possibly by excluding water that would hydrolyze the donor substrate, or to form a lid over the nucleotide binding site allowing acceptor to bind, or to allow movement, and facilitating the reaction.

Generally, GTs have a distinct acceptor substrate specificity and with few exceptions, utilize only one type of nucleotide sugar donor substrate. Although few of the bacterial GTs have been biochemically characterized, it appears that both bacterial and mammalian GTs generally have similar properties with respect to their optimal pH, metal ion requirement, and donor specificity, although some bacterial GTs have a more promiscuous acceptor specificity (59). They can, thus, synthesize unnatural linkages that may find application as inhibitors or for biological studies. For example, β4-Gal-transferase LgtB from *Helicobacter pylori* (*Hp*) has been used to synthesize thio-glycosides.

A comparison of mammalian and corresponding bacterial GTs (Table 2) shows that there is a low percentage of amino acid identity (often <12%), although the activities are comparable and the sugar transfer reactions follow similar mechanisms. Exceptions are ABO transferases, GTA and GTB, that synthesize blood group A and B, respectively, similarly in human beings and in certain bacteria, and show about 20% identity. Some of the α2 and α3/α4-Fuc-transferases also have similar activities when comparing human and bacterial GTs and show 14.5–17.5% identity. This suggests an exchange of genes between mammals and bacteria or a common evolutionary origin. The similarity and identity between GTs with similar function in bacteria or within the eukaryotic GT families can be much higher. The arrangements of amino acids in the catalytic site may therefore be similar, leading to the binding of the same nucleotide sugars and acceptors with the transfer of the sugar in a specific linkage. The requirement of a metal ion to stabilize the negative charge of the nucleotide sugar may also be the same. An evolutionally conserved feature of GTs is that the catalytic mechanism usually involves a catalytic base.

Inverting GT (52, 57, 58) inverts the anomeric configuration of the sugar in the donor substrate. This inversion is expected to follow a single displacement where the catalytic base deprotonates the hydroxyl group of the acceptor to be glycosylated, which then becomes an active nucleophile attacking carbon-1 of the sugar of the donor substrate. This mechanism involves an S_N_2 reaction and an oxocarbenium ion transition state. Crystal structures show that the catalytic base (Asp, Glu, or His) is properly positioned near the hydroxyl to be glycosylated. In many cases, this catalytic residue is within a conserved DxD motif (Table 5). Both the DxD motif and the negatively charged phosphate group of the nucleotide leaving group may be stabilized by a divalent metal ion, but positively charged amino acids could also serve this function (20). Inverting GTs have been shown to act with a sequential ordered mechanism.

**Table 5:** **Conserved peptide motifs in glycosyltransferases**.

	Peptide motif	Reference
Many GTs	DxD	(60)
Beta4-Gal-transferases	DxD, GWGxED	(60, 61)
Beta3-Gal-transferases	DxD, 2 motifs	(61)
Fuc-transferases	2-Fuc motif	(60, 62, 63)
	Two 3-Fuc motifs	(60, 62, 63)
	6-Fuc motif	(60, 62, 63)
	Three 2/6-Fuc motifs	(60, 63, 64)
	HxRRxD	
Beta3-GlcNAc-transferases	DxD, 2 motifs	(61)
C2GnT	SPDE	(20, 21)
Sialyltransferases, mammalian	Sialyl-motif L	(65)
	Sialyl-motif S	(65)
	Sialyl-motif VS	(65)
	Sialyl-motif III	(65)
Sialyltransferases, bacterial	D(E)-D(E)-G	(66)
	HP	
Polysialyltransferases, mammalian	PSTD motif	(67)
Polypeptide GalNAc-transferases	Ricin-like lectin domain	
ABO transferases	DxD	(60)

GTs that retain the anomeric linkage of the nucleotide sugar may function in a double displacement mechanism (58). Thus, for retaining GTs, a short-lived glycosyl-enzyme intermediate may form. This is followed by a shift in protein conformation that allows a nucleophilic attack on the anomeric center of the sugar by the deprotonated hydroxyl of the acceptor substrate to be glycosylated, maintaining the original anomeric linkage. A double displacement mechanism has been proposed for GalNAc-transferase GTA and Gal-transferase GTB, and a covalent glycosyl-enzyme intermediate through Cys303 was found (68). Other mechanisms may be possible and need to be investigated for retaining enzymes (58). GTs can also transfer sugar to water and thus have a nucleotide sugar hydrolase activity.

Mammalian GTs are single or multiple membrane-spanning proteins in the ER or single transmembrane-spanning type II membrane proteins in the Golgi, with a short cytosolic domain, a transmembrane anchor domain, and a stem region that helps the globular catalytic domain to protrude into the Golgi lumen. In the bacterial inner membrane, the first enzyme that adds the sugar-phosphate to P-Und such as WecA, as well as related sugar-phosphate transferases has multiple membrane-spanning domains. The remaining bacterial GTs that assemble O antigen repeating units do not have a transmembrane domain but have short hydrophobic stretches that may contribute to an association with membrane components. It is possible that both, mammalian and bacterial GTs, exist in protein/membrane complexes that activate enzymes and make the assembly of glycan chains highly efficient.

## A Large Family of Gal-transferases

Families of at least 5 β3-Gal-transferases (B3GALTs) and at least 7 β4-Gal-transferases (B4GALTs) participate in forming the extensions of glycoproteins (69) that are the basis for the attachments of epitopes including the Lewis^x^ antigen, the selectin ligand involved in the inflammatory response (8). These inverting metal ion-dependent GTs have a DxD motif, bind UDP-Gal and a number of GlcNAc-terminating acceptor substrates.

### The B4GALT family

The crystal structures of both, human and bovine β4-Gal-transferases 1 (B4GALT1) in complexes with donor and acceptor substrates and several mutants, have been thoroughly studied (22). UDP-Gal binds in a deep catalytic pocket of the bovine B4GALT1 together with Mn^2+^, in the vicinity of Asp252, Asp318, and Glu317 residues. The conformational change induced by binding UDP-Gal creates the binding site for GlcNAc-terminating oligosaccharides. The GlcNAc moiety, which needs to be in the β-anomeric configuration is bound by Phe280, Phe360, Tyr286, Arg259, and Ile363. The enzyme has three DxD sequences.

In the bovine B4GALT1 enzyme, the first Asp254 residue in the DVD motif has contact with UDP and Mn^2+^ but mutations of Asp318 or Asp320 within the DDD sequence show that these residues are essential for activity. His344 normally interacts with Mn^2+^. A His344Met mutant is active in the presence of Mg^2+^, instead of Mn^2+^ and maintains a closed conformation bound to Mg^2+^ and UDP-hexanolamine, allowing an acceptor to bind. The mutant is, thus, useful to study the role of conformational changes and the binding of various acceptors (70, 71).

The catalytic domain of B4GALT1 has a short and a larger flexible loop containing the metal ion binding site. The binding of the donor and metal ion induces conformational changes in the long flexible loop, which changes from the open to the closed conformation, creating a lid over the bound nucleotide sugar. This opens an acceptor-binding site at the C terminus of the flexible loop. After the transfer reaction, the loop changes back to the open conformation, releasing the nucleotide (72). β4-Gal-transferase 7 (B4GALT7) is another member of the same family, involved in priming glycosaminoglycan synthesis by adding Gal to Xylose (24). B4GALT7 also works in an S_N_2 type mechanism and changes conformation from closed to open conformation upon binding UDP and Mn^2+^. The mammalian β4-Gal-transferases have a common B4GALT motif GWGxED, which is not found in β3-Gal-transferases or in the bacterial counterparts of B4GALT (61) (Table 5).

β4-Gal-transferases that synthesize Galβ1-4GlcNAc sequences are also found in bacteria. For example, β4-Gal-transferase LgtB from *Helicobacter pylori* (*Hp*) can synthesize Galβ4-S-GlcNAc and Galβ4-Man linkages (59). The repeating unit of *Shigella boydii* (*Sb*) also contains the Galβ1-4GlcNAc sequence, which is synthesized by β4-Gal-transferase WfeD (73). The sequences of human β4-Gal-transferase and WfeD have about 9% identity; yet, the reaction catalyzed is similar. Both enzymes are inverting GTs, bind UDP-Gal and GlcNAc-R acceptor substrates, are activated by Mn^2+^, and have a DxD motif. Interestingly, we found that both enzymes are also activated by Pb^2+^, although the activation of the bacterial enzyme is much higher and is similar to Mn^2+^ activation. While human β4-Gal-transferase is in the GT7 family with a GT-A fold, the structure and predicted fold of the WfeD in GT family 26 is uncertain (Table 3). The human enzyme does not accept the negatively charged bacterial acceptor substrate, GlcNAc-PP-lipid, and vice versa, the bacterial enzyme cannot act on GlcNAcβ-Bn, which is the standard acceptor for assays of the human enzyme. Mutagenesis of WfeD showed that the central Glu101 residue of the DxExE sequence is essential for activity. Lys211 was also found to be important, possibly by binding one or two phosphate group(s) of the acceptor substrate (73). Lys residues are apparently not involved in catalysis of the human enzyme. WfeD is not inhibited by GlcNAcβ-naphthyl, which is a potent inhibitor of the mammalian β4-Gal-transferase (74).

### The family of β3Gal-transferases (B3GALT)

Human glycoproteins can be extended with Galβ1–3GlcNAc (type 1) sequences that are also found in O antigens, e.g., in the repeating unit structure of the *E. coli* O7 antigen. There are five enzymes that synthesize the Galβ1–3GlcNAc linkage on a variety of acceptors in mammals. They are inverting GTs having a DxD motif and a requirement for divalent metal ions such as Mn^2+^ (15, 69). B3GALT5 has a distinct specificity for O-glycan core 3 (GlcNAcβ1–3GalNAc-) acceptors. However, crystal structures are not available for β3-Gal-transferases. Members of the β3-Gal-transferase family have two common peptide motifs, in addition to the DxD motif (Table 5).

A β3-Gal-transferase WbbD from *E. coli* O7 was detected that can act on GlcNAcα-PP-lipids where apparently the lipid structure is of minor contribution to the activity (75). The enzyme belongs to the GT2 family with a predicted GT-A fold and synthesizes the disaccharide Galβ3GlcNAc α-linked to PP-lipid as the second step in repeating unit synthesis. Deletion of the enzyme eliminates the synthesis of O antigen on LPS. This supports the idea that an inhibition of this second step is successful in creating bacteria that are more susceptible to the mammalian immune system.

### Biosynthesis of the Thomsen–Friedenreich (TF) antigen

The cancer-associated T antigen, Galβ1–3GalNAc-, core 1, is the precursor for most O-glycans. In cancer, core 1 is often found in the unsubstituted form, while in normal glycoproteins, it is substituted by other sugars and is thus not recognized by anti-T antibodies. Sialylation of core 1 is also common in glycoproteins and often overexpressed in cancer and is recognized as the sialyl-T antigen (15). Several bacteria carry the T antigen as an internal structure within their O antigen repeating unit. The Shiga toxin producing O104 serogroup of *E. coli* is unusual in that it contains the T antigen in its O antigen repeating unit, as well as the sialyl-T antigen, sialylα2–3Galβ1–3GalNAc- (ECODAB).

The core 1 structure in human beings is synthesized by core 1 β3-Gal-transferase (T synthase, C1GALT1) and deficiencies of the enzyme are associated with pathological conditions including cancer. T synthase is the only known GT that requires the co-expression of a chaperone protein, Cosmc, C1GALT1C1 (76). C1GALT is a GT31 family member with a predicted GT-A fold, requires Mn^2+^ for activity and prefers GalNAcα-glycopeptides as substrates but can also transfer Gal from UDP-Gal to GalNAcα-benzyl and related substrates (77).

The GTs responsible for the synthesis of the T antigen in bacteria have a similar function (Table 2). The T synthase WbwC in the *E. coli* O104 strain is within the GT2 family (Table 3), and has only 10.5% identity compared to human C1GALT. No chaperone is necessary for the expression and activity of the bacterial enzyme (78). Both, human C1GALT and WbwC have a GT-A fold and DxD motifs, utilize UDP-Gal as a donor and require Mn^2+^ as a cofactor. However, in contrast to C1GALT, WbwC has a specificity for GalNAcα-diphosphate-lipid acceptor, while GalNAcα-peptides are not substrates. At this time, no crystal structure is available for T synthases but it is conceivable that the three-dimensional amino acid arrangements in the catalytic sites are similar. WbwC and human C1GALT could be distinguished using bis-imidazolium salt inhibitors, which showed that only WbwC, but not human C1GALT, was strongly inhibited with IC_50_ values of 8 μM (78). These inhibitors could selectively attack GTs in pathogenic bacteria. However, a potent inhibitor for T synthase has yet to be discovered (77).

### P blood group synthesis

Human blood group P (Table 1) and related, complex structures containing the Galα1–4 linkage are synthesized by α4-Gal-transferases (A4GALT), mainly using glycolipids with Gal residues as acceptors, e.g., lactosylceramide (79). However, a different α4-Gal-transferase from pigeon, related to β4-Gal-transferase from the same species, but not to β4-Gal-transferases from human beings, has been described that preferably acts on the N-glycans of glycoproteins (80).

A number of bacteria, including *Cj* (81), also express an α4-Gal-transferase with about 11% identity to the human enzyme (Table 2). The LgtC α4-Gal-transferase from *Nm* synthesizes the bacterial mimic of the human P blood group (45). The enzyme is a member of the GT8 family with a GT-A fold and follows a bi-bi kinetic mechanism where UDP-Gal binds first. The crystal structure of LgtC with analogs of UDP-Gal and lactose substrates suggests that Asp103 and Asp105 of one of the four DxD motifs, as well as His244, are in the vicinity of the donor substrate, while a Mn^2+^ ion coordinates the phosphates of UDP. The mainly helical C terminus is expected to form hydrophobic and electrostatic interactions with the bacterial membrane. Multiple conformational states of LgtC with and without bound substrate analogs were found by methyl-TROSY NMR (82), which is additional information that cannot be obtained by static crystal structure analysis.

### A new DxDD motif in GT2 transferases

A new DxDD motif (Table 5), essential for activity, was discovered in WbwC (78). This motif is also present in WbdN, WfaP, WfgD, WbgO, WbiP, and CgtB (83–87). All of these GTs in the GT2 family having a DxDD motif are specific for the transfer of either Gal or Glc in β1–3 linkage to GalNAc or GlcNAc. Mutagenesis showed that in WbiP from *E. coli* O127 (83), the first Asp of the DxDD sequence was critical for activity while the second contributed but was not essential. In WbwC from *E. coli* O104 and O5, all three Asp residues were mutated and found to be important for activity. The first Asp (D91) is probably the catalytic base. The other Asp residues may support the nucleophilic property of the catalytic base (78).

While WbwC synthesizes the Galβ1–3 linkage attached to the first GalNAc residue at the reducing end of the O antigen repeating unit, several other GTs having a DxDD motif in the GT2 family were shown to synthesize the T antigen at a more internal position of the repeating unit. These GTs have a different specificity from that of WbwC and do not require the diphosphate in the acceptor. The T synthase activities of variants of CgtB from *Cj* mainly act on β-linked GalNAc acceptors. Variants of CgtB have distinct acceptor specificities (86) and synthesize lipooligosaccharides, which mimic mammalian glycolipids and glycoproteins.

## GlcNAc-Transferases Form Backbone Structures

Gal-transferases cooperate with five or more β3-GlcNAc-transferases (B3GNT) within the GT31 family to form the type 1 and 2 backbone structures of mammalian glycan chains (15, 88, 89) (Table 3). B3GNTs have significant sequence similarity with Gal-transferases. It is not known if these enzymes are physically associated, although their combined action would suggest this. A family of three β6GlcNAc-transferases (IGnT, GCNT2) then can add 1–6 branches to the linear chains. The β3-GlcNAc-transferases, but not the β6-GlcNAc-transferases, require divalent metal ions for activity. No crystal structures are yet available for B3GNTs.

In the N-glycosylation pathways, GnT I to V (MGAT1 to 5) (12) are responsible for forming GlcNAc-based antennae that can be further extended through repeating linear or branched GlcNAcβ1–3Gal-disaccharides. MGAT1 is an inverting GT with a GT-A fold within the GT13 family. The crystal structure of rabbit GnT I with UDP-GlcNAc and Mn^2+^ supports an ordered sequential mechanism. The DxD motif is present as EDD, with Glu211 being the likely catalytic base (19). MGAT2, 3, 4, and 5 are all inverting GTs and have been classified in the GT16, GT17, GT54, and GT18 families respectively. Although the GT17 family also contains uncharacterized bacterial proteins, no bacterial equivalents of MGAT have been found in bacteria.

In the O-glycosylation pathways, the basis of most extended chains is core 2. Core 2 β6-GlcNAc-transferase C2GnT1 (GCNT1) adds a branch to O-glycan core 1 to form the core 2 structure GlcNAcβ1–6(Galβ1–3)GalNAc-R (15). The enzyme has a GT-A fold and is classified in the GT14 family. The crystal structure of the catalytic domain of mouse C2GnT1 shows that the protein has four conserved intramolecular disulfide bonds (20, 21). Cys217, however, has to be reduced to support the activity, although it is not an essential residue (90). The human enzyme expressed in insect cells has two flexible N-glycans that protect the protein from degradation (91). C2GnT1 is an inverting GT that is active in the presence of EDTA and does not require Mn^2+^. The crystal structure suggests that the conserved, basic amino acids Arg378 and Lys401 stabilize the diphosphate group of UDP-GlcNAc and thus serve the function of Mn^2+^. The structure supports specificity studies of C2GnT1, showing an absolute requirement for the 4- and 6-hydroxyl groups of the Gal and GalNAc residues and the 2-acetamido group of GalNAc (77). Glu320 of the conserved SPDE sequence may be the catalytic base; it binds to the 4 and 6-oxygen of GalNAc and could thus deprotonate and activate the 6-hydroxyl to induce a nucleophilic attack on the C-1 of the GlcNAc moiety of UDP-GlcNAc (20, 21).

Bacteria do not appear to have C2GnT or GnT I equivalents, but they express type 1 of type 2 chains and β3-GlcNAc-transferases comparable to the mammalian enzymes in their activities. For example, a β3-GlcNAc-transferase from *Hp* is involved in the synthesis of lipooligosaccharides and GlcNAcβ1–3Gal- extensions that resemble mammalian epitopes (92). The β3-GlcNAc-transferase LgtA from *Nm* acts on lactose and has a relaxed donor specificity. It is most active with UDP-GlcNAc but can also utilize UDP-GalNAc (93). Both, the mammalian and bacterial β3-GlcNAc-transferases accept a wide variety of acceptor substrates but have low sequence identity (Table 2) (15).

## Fucosyltransferases

Three different types of Fuc-transferases (FUT) are involved in glycoprotein biosynthesis. Peptide motifs have been identified that are specific for α2-Fuc-transferases, α3-Fuc-transferases, or α6-Fuc-transferases or are shared by α2- and α6-Fuc-transferases (62). The mammalian α3-Fuc-transferases (FUT3-7,9-11) are inverting enzymes (Table 3) and have two shared motifs with similar spacing, shared by eukaryotic and bacterial α3FUT. The α2-Fuc-transferases FUT1 and 2 have less than 30% sequence identity with their bacterial FUT counterparts, but have well-preserved α2-Fuc-transferase motifs. There are also similarities between α2- and α6-FUT (FUT8) (60) with three common motifs (I to III), shared among eukaryotic and bacterial enzymes (62). The different types of FUT may have evolved from a common ancestor by divergent evolution (63).

### Fucosyltransferases that synthesize the H antigen

The blood group O (H antigen, Fucα1-2Gal-R) is found in virtually all human beings and in certain bacteria and is the precursor substrate structure to form blood groups A and B. The enzymes that synthesize the H antigen in human beings are inverting α2-Fuc-transferases 1 and 2 (FUT1 and FUT2) that are closely related in sequence to the GT6 family ABO transferases GTA and GTB, although FUT1 and 2 have been classified into a different (GT11) family. FUT1 has a broad acceptor specificity for Galβ-R while FUT2 prefers O-glycan core 1 (T antigen) as a substrate (15).

Similar enzymes (Table 2) have been identified in *Hp* as FutC (94), in *E. coli* O86 as WbwK (95), as WbsJ in *E. coli* O128 (64), and WbiQ in *E. coli* O127 (96). WbwK and WbiQ have a distinct specificity for the T antigen (95, 96) and do not act on Galβ1–4 glycans. These FUT, therefore, have an activity resembling that of human FUT2 and have 12–17.5% sequence identity. HpFucT2 (FutC) adds Fuc preferably to Lewis x acceptors but also uses Lewis a and type 1 chains (94). In contrast, WbsJ prefers acceptors with terminal Galβ1-4Glc structures (64). WbsJ functions in the absence of divalent metal ion and does not have a DxD motif. Especially the first Arg residue of the HxRRxD motif, conserved in α2- and α6-Fuc-transferases, is critical for activity due to its positive charge. Domain swapping between WbwK and WbsJ showed that the C-terminal motifs function in determining acceptor specificity (95). All of the identified α2-Fuc-transferases have significant homology in GT family 11 (Table 3) with a predicted GT-B fold but none have been crystallized.

### Fucosyltransferases involved in the synthesis of Lewis antigens

Lewis type antigens play essential roles in cell adhesion in the immune system and during inflammation, and aberrant amounts are often found in cancer. A family of mammalian, inverting α3-Fuc-transferases (FUT3–7, 9–11) is involved in Lewis antigen synthesis by linking Fuc to GlcNAc (9, 15, 97, 98). The enzymes vary in their acceptor substrate specificities and cell type expression and are in the GT10 family with a GT-B fold. FUT3 is an exceptional enzyme that has a dual specificity and adds Fucα1–3 on type 2 chains to synthesize Lewis x and y, as well as Fucα1–4 to type 1 chains to synthesize Lewis a and b (Table 1). FUT5 also shows some α4-Fuc-transferase activity. Human FUT3 and 5 have Trp111, responsible for type I acceptor recognition and 1–4 linkage synthesis. FUT that do not have this Trp synthesize the 1–3 linkage (99).

The bacterial α3FUTs show weak homology to mammalian FUT in two small segments of the catalytic domains (α3FUT motifs). They have about 10% sequence identity and a common GT-B fold but no transmembrane domain (25, 62). Two amphipathic α-helices serve to anchor the enzymes in the membrane. The gastric pathogen *Hp* is a prime example of expressing human-like type 1 and type 2 chains that are fucosylated and include Lewis antigens, which may play a role in adhesion to gastric epithelial cells or in internalization. *Hp* have short O antigens (lipooligosaccharides) and the human glycan mimics help to mask the immunogenic determinants of *Hp*, thus evading immune surveillance and supporting persistent *Hp* infections. The different pH environments in the various regions of the stomach influence the expression of Lewis antigens, and likely the activities of GTs, leading to phase variations.

A number of bacteria have Fucα1–4 linkages but *Hp* is especially rich in Lewis a, b, x, and y structures and in α3/4-Fuc-transferase activities (100). All of the eukaryotic and most bacterial α3-Fuc-transferases are in the GT10 family. *Hp* has futA and futB genes encoding α3FUT, in addition to 1-3/4 FUT (FucTa). FucTa has the CNDAHYSALH sequence near the C terminus that controls type I chain recognition. It seems that in this α3/4 FUT, it is Tyr instead of Trp that determines the acceptor preference. Thus, the Y350A mutant synthesizes Lewis x since it had dramatically reduced α4 FUT activity (100).

The crystal structure of α3-Fuc-transferase from *Hp* shows that a Glu95 residue is positioned closely to the anomeric carbon of Fuc of the donor GDP-βFuc and could be a catalytic base (25) while Glu249 could stabilize the intermediate oxonium ion. Mutants in these Glu residues are virtually inactive. Interestingly, tandem repeats of 7 amino acids (DDLRINY) are found in this α3FUT. The 2–10 heptad repeats appear to connect the N terminus to 2 amphipathic helices at the C terminus and are thought to be involved in maintaining secondary structure and activity (101). The C terminal sequence appears to determine the stability and overall structure of the protein.

A different α3-Fuc-transferase HhFT2 from *Helicobacter hepaticus* (*Hh*) synthesizes the Lewis x as well as the sialyl-Lewis x antigen (102). This enzyme is a member of the GT11 family and has more homology to α2-Fuc-transferases such as WbsJ of GT11, but less to alpha3/4 FUT in GT family 10. It has 10.4% sequence identity with the human enzyme FUT4. HhFT2 has three conserved motifs, one at the N terminus, one central, and one near the C terminus (Table 5).

### Synthesis of the Fucα1-6GlcNAc Linkage

The α6-Fuc-transferases add Fuc in α1–6 linkage to the reducing end GlcNAc of the N-glycan core. The human enzyme (FUT8) requires the prior action of GnT I and cannot act when the chitobiose of the N-glycan core carries an α3Fuc residue, or if the internal Manβ residue carries the bisecting GlcNAc. FUT8 is classified in family GT23 with a GT-B fold. The crystal structure of human FUT8 shows three domains: an N-terminal coiled-coil domain, a catalytic domain that resembles GT-B folded GTs, and a C-terminal SH3 domain, although its significance is unknown. The C-terminal part of the catalytic domain contains a Rossmann-like fold with three regions, conserved in α2-, α6-, and other Fuc-transferases. Both Arg365 and Arg366 are critical for binding to GDP-Fuc while Asp453 may be a critical catalytic base (26).

A bacterial α6-Fuc-transferase with similar activity in the GT23 family with a GT-B fold and only 8% sequence identity is NodZ from *Rhizobium* sp. *(Rsp)* (Tables 2 and 4) (103). The crystal structure of NodZ shows two domains of nearly equal size but with different shape, separated by a central cleft (27). There are three conserved sequence motifs near the C terminus that play a role in GDP-Fuc binding or catalysis.

## Glycosyltransferases that Synthesize Blood Groups A and B

The two human ABO transferases that synthesize the antigenic blood group A and B determinants from the H antigen (α3-GalNAc-transferase GTA and α3-Gal-transferase GTB, respectively) are homologous retaining enzymes within the GT6 family with a GT-A fold (Table 3). It is astounding that the critical difference in donor specificities determining blood group A or B lies in a difference of only four amino acids. While GTB that transfers Gal has Gly176, Ser235, Met266, and Ala268, the GTA protein that transfers the slightly larger GalNAc has mostly smaller amino acids Arg176, Gly235, Leu266, and Gly268.

In GTA and GTB, two domains are separated by a catalytic cleft containing the DxD motif (Asp211–Asp213) (39). However, a highly conserved Glu303 is likely to be the active nucleophile. UDP binds in the nucleotide sugar-binding domain at the N terminus and the Mn^2+^ ion coordinates the β-phosphate of UDP. The H antigen acceptor binds to the C terminus. A disordered and flexible internal loop adjacent to the active site (40) becomes ordered when the nucleotide (sugar) is bound. This leads to a conformational change in the protein (43). Two amino acids are in contact with donor or acceptor in GTA and GTB (39) but only one of them determines the binding of the nucleotide-bound sugar moiety, i.e., either Gal or GalNAc. Leu266 in GTA has contact with the acetamido group that allows binding of UDP-GalNAc. Due to the larger Met in this position (Met266), GalNAc cannot be accommodated and, therefore, Gal binds. Ala/Gly268 has contact with the 3- and 4-hydroxyl groups of Gal and thus does not contribute to the difference in donor specificity.

Human beings have antibodies against the absent blood group (A or B), and it is possible that this is induced by bacteria displaying this blood group. A number of bacterial GTA-like enzymes are also in the GT6 family and resemble the human counterpart with relatively high sequence identity of about 20%. The similarities between human and bacterial enzymes suggest a horizontal gene transfer between species and between bacteria. The bacterial enzymes have an NxN sequence instead of the eukaryotic DxD motif, and most of these enzymes do not have a metal ion requirement. Thus, bacterial enzymes may have altered catalytic mechanisms, although there is a strong conservation of mammalian-type of residues in the active sites (104).

*Helicobacter mustelae* (*Hm*) synthesize the blood group A determinant, which reacts with anti-human blood group A antibodies (105). The enzyme responsible, GTA-like α3-GalNAc-transferase (BgtA), has 20% sequence identity to its human counterpart GTA and can act on Fucα1-2Galβ1–3-R or Fucα1–2Galβ1–4-R substrates (Table 2). Thus, bacteria may have acquired the GTA gene from a mammalian host, enhancing their molecular mimicry, although it is not clear how the human blood group is giving them a selective advantage.

The GTA-like enzyme BoGT6a from *Bacteroides ovatus* (*Bo*) (44) and GTB-like α3-Gal-transferase WbnI from *E. coli* O86 (95) that synthesize blood group B are related to the human enzymes with significant sequence homology in the GT6 family. Both donor and acceptor substrates are the same as those for GTA and GTB from human beings. The crystal structure of BoGT6a revealed a disordered region, which becomes ordered when acceptor Fuc-lactose is bound. This is accompanied by a large conformational change from the open to a closed state. Isothermal titration calorimetry (ITC) experiments showed that BoGT6a binds UDP-GalNAc with high affinity.

In non-primate mammals and new world monkeys, the linear blood group B occurs (Galβ1–3Gal-), without the α2-linked Fuc residue. This structure is foreign to human beings who have anti-linear B antibodies, thus hindering xenotransplantation. The α3-Gal-transferase A3GALT that synthesizes the linear B determinant is a homolog of GTA and GTB and has been crystalized with UDP and Mn^2+^ (41). The invariable Glu317 was identified as the catalytic base. The crystal structure in a complex with Galβ-pnp suggests that Trp residues are critical for binding the natural substrate Galβ1 -4GlcNAc (106). The disordered C terminal region is critical for allowing the substrate to bind (42). Bacterial analogs of this α3-Gal-transferase remain to be characterized.

## Sialyltransferases in Mammals and Bacteria

Sialyltransferases are ubiquitous in eukaryotes and are also expressed in certain bacteria (107). These enzymes synthesize sialic acid linkages commonly found on the non-reducing termini of N- and O-glycans, and gangliosides as sialylα2–3Galβ1-or sialylα2–6Gal(NAc)-linkages. In addition, sialylα2–8 linkages are found, especially in polysialic acids (PSA), which are extremely large, linear polymers, expressed in a cell type specific, restricted fashion in embryonic, neuronal, and other selected cell types (108). Sialic acids contribute to the acidity and hydration of a glycoprotein, the metal ion binding, and epitope exposure. While sialic acid can mask the underlying epitope, certain lectins of the immune system (e.g., siglecs) directly recognize sialic acid in specific linkages. Metastatic cancer cells and leukemia cells are often hypersialylated, which reduces further processing of glycans and causes glycan chains to be shorter (15). Sialylation significantly affects the adhesive properties of cells and has also been implicated in the functions of cell surface receptors (109).

Sialyltransferases are inverting GTs that usually lack a DxD motif and do not require divalent metal ions. Thus, general acids and bases identified in the crystal structures of α3- and α6-sialyltransferases that may interact closely with the substrates include His residues (28, 33). All known eukaryotic sialyltransferases have been classified as inverting GT29 with a GT-A fold, having at least four sialylmotifs (Table 5), a large (L), small (S), very small (VS), and motif III (65). The L motif contains the donor binding site while the S motif also binds the acceptor.

Bacterial sialyltransferases are inverting enzymes that bind CMP-sialic acid donor substrate and acceptors terminating in Gal or sialic acid but do not have these sialylmotifs and do not belong to the GT29 family. Instead, they are classified as GT42 (with a GT-A fold), GT52 or GT80 (with a GT-B fold), and GT38 (polysialyltransferases, PSTs). The 6-sialyltransferases (ST6GalNAc) acting on O-glycans do not appear to have a bacterial counterpart. Two highly conserved short motifs have been identified in bacterial PST and other bacterial sialyltransferases (GT52 and GT80), a C-terminally located HP sequence and a more N-terminally located D/E-D/E-G sequence (66). Certain bacterial sialyltransferases have multiple activities, including CMP-sialic acid hydrolase, trans-sialidase, and neuraminidase activities and are usually from the GT80 family. Thus, sialyltransferases can be promiscuous with respect to the linkages they form (or cleave) and the acceptor substrates they recognize. Bacterial sialyltransferases probably evolved separately from the eukaryotic enzymes, although their functions and mechanisms can be similar.

### Alpha3-sialyltransferases

In human beings, 6 α3-sialyltransferases (ST3GAL) form the sialylα2–3 linkage. The expression and activity of α3-sialyltransferase ST3GAL1 that synthesizes sialyl-T antigen are increased in breast cancer (110) and appear to promote survival of cancer cells in the blood (111). In keeping with its activity in adding a terminal structure, ST3GAL1 is localized to the medial and late Golgi compartments in human mammary cells (112). ST3GAL1 acts on glycopeptides with core 1 structure and also on Galβ1–3GalNAcα-R acceptors that have hydrophobic aglycone groups. In contrast, a bacterial equivalent, WbwA from *E. coli* O104, responsible for the rare occurrence of the sialyl-T antigen in *E. coli*, is in the GT52 family with a GT-B fold. WbwA, but not mammalian ST3GAL1, also has HP and D/E-D/E-G motifs (Table 5). The crystal structure of porcine ST3GAL1 with CMP and Galβ1–3GalNAc-acceptor substrate suggests that the essential His302 interacts with the phosphate of CMP-sialic acid. His319 is the catalytic base in motif III that is proposed to be positioned near carbon-2 of the sialic acid moiety (28). The conserved Tyr269 residue interacts with the 4-hydroxyl of GalNAc and thus determines the enzyme specificity for Galβ1–3GalNAc- over the Galβ1–3GlcNAc- acceptors.

An α3-sialyltransferase of the GT80 family from *Photobacterium Phosporeum* (*Pp*) has been crystallized with CMP (29). The acceptor-binding site has a wide access explaining that a range of possible disaccharides with Galα and Galβ linkages can form substrates. CMP binds in a cleft between the two domains of the GT-B fold. The main chain nitrogen of His317 in the HP motif is close to the nitrogen-4 of Cytidine and the side chain of His317 is near the oxygen of the phosphate. This suggests a critical role of these His residues in catalysis. Another α3-sialyltransferase with a GT-B fold in family GT52 from *Nm* was crystallized (31) with the donor analog CMP-3F-Neu5Ac. Asp258 could be a general base and His280 (within the HP motif) a general acid.

### Alpha6-sialyltransferases

Human α6-sialyltransferases add sialic acid to the Gal termini of N-acetyllactosamine chains of N-glycans. ST6GAL1 is highly expressed in colon cancer and metastatic cells (113) and also resides in the trans-Golgi (114). A homolog with 48% sequence identity (ST6GAL2) is mainly expressed in the brain and has the additional ability to synthesize sialylα2–6GalNAcβ1–4GlcNAc- structures (115). Human ST6GAL1 is a glycoprotein stabilized by three disulfide bonds (33). The catalytic residue, His370, deprotonates the 6-hydroxyl of Gal, generating an active nucleophile that attacks the carbon-2 of sialic acid. The reaction follows a random-order mechanism of substrate binding. Rat ST6GAL has three disulfide bonds and two N-glycans (32). As many GTs, the enzyme has a disordered loop, and His367 is the catalytic base within the sialyl motif VS.

The bifunctional bacterial α6-sialyltransferase PM0188 from *Pasteurella multocida* (*Pm*) of GT family 80 has 14.6% sequence identity to human ST6GAL1. The crystal structure showed the GT-B fold and that Asp141, His311 (within the HP motif), Glu338, Ser355, and Ser356 were important for catalysis (37). The *Photobacterium* sp. *(Psp)* α6-sialyltransferase was also crystallized with CMP and lactose (35). The enzyme is in the GT80 family with a GT-B fold and has three domains, with the donor and acceptor bound between domains 2 and 3. Asp232 (within the D/E-D/E-G motif) is near the 6-hydroxyl of Gal while the nitrogen of His405 (within the HP motif) is close to the phosphate-oxygen. Thus, Asp232 could be a catalytic base that deprotonates the 6-hydroxyl of Gal, and His405 could be a catalytic acid that protonates the donor substrate.

### Multifunctional sialyltransferases

In bacteria, mimics of human sialylα2-3/6/8Galβ1- structures occur, e.g., in the lipooligosaccharides of Gram-negative bacteria such as *Cj* (116, 117). Cells of the human nervous system are rich in gangliosides as well as glycoproteins containing similar sialyl-linkages. Thus, after bacterial infections, cross reactivity of antibodies could cause the rare development of neurological disorders. Guillain–Barré syndrome is an example (118). *Cj* expresses a bifunctional α3/8-sialyltransferase CstII and an α3-sialyltransferase CstI, which are responsible for the molecular mimicry of *Cj* in their lipooligosaccharide structures. Both enzymes have a predicted GT-A fold within the GT42 family. The structure of CstI shows (30) that His 202 is the catalytic base. Similarly, His188 is likely the catalytic base in CstII that deprotonates the 3-hydroxyl of Gal, which then attacks carbon-2 of sialic acid of the donor (119). The flexible lid in the CstII protein becomes ordered in a closed form when CMP binds (36). The acceptors lactose (for α3-sialyltransferase activity) or sialyl-lactose (for α8-sialyltransferase activity) bind in a cleft and Arg129, Asn51, and Tyr81 contribute to the binding of the sialylated acceptor. The role of His188 as a catalytic base in CstII has also been confirmed by NMR studies (120). The intrinsic *pK*_a_ values of His188 were measured in monomeric mutants by determining the pH-dependent chemical shifts of [^13^C]-labeled His188.

The monofunctional sialyltransferases function with similar mechanisms compared to the mammalian enzymes. Multifunctional enzymes, however, are primarily found in bacteria and include the α3-sialyltransferase PmST1 from *Pm*, which binds CMP-sialic acid as donor and lactose, Gal, GalNAc as well as sialic acid as acceptor. The crystal structure shows that binding of CMP-sialic acid donor substrate causes a change in conformation and opens the acceptor-binding site. The activities of PmST1 function optimally at different pH values. It has a GT-B fold within the GT80 family (Table 3). The crystal structure shows that Asp141 is the catalytic base (34) with His112 also being important for enzyme activity. Another multifunctional α3-sialyltransferases PdST from *Pasteurella dagmatis* (*Pd*) in the GT80 family with a GT-B fold is also a CMP-sialic acid hydrolase. At low pH, it can act as a trans-sialidase and a sialidase (121).

### Polysialylation

Important sialic acid structures are the PSA, found in human neuronal and other selected cell types (107). Only a selected number of proteins carry the PSA modification (122). For example, polysialylated neural cell adhesion molecule N-CAM is prominent in the developing nervous system but also occurs in leukocytes with roles in the regulation of cell adhesion. N-CAM becomes anti-adhesive when long polymers of α2–8-linked sialic acids are covalently attached to its N-glycans (108). The sialylα2–8 linkages of PSA are synthesized based on sialylα2–3/6Gal residues of N-glycans by developmentally regulated PSTs, which are highly expressed in the developing and embryonic brain (123). Neuropilin-2 (NRP2) is a glycoprotein containing multiple N-glycosylation sites, as well as O-glycans with sialylated core 1 and 2 structures. In cells lacking core 2, human PST (ST8SiaIV) was shown to assemble PSA on sialylated core 1 chains of neuropilin (124). The presence of these PSA polymers extends the half-life of proteins.

*E. coli* and *Nm* are examples of bacteria that carry sialylα2–8 polymeric PSA capsules, which help bacteria to resist phagocytosis. These PSA capsules mimic the eukaryotic chains, although they are linked to the membrane via a lipid anchor, and may have bacteria-specific modifications such as O-acetylation (125). The large, charged and hydrated polymeric enzyme product is assembled in the cytoplasmic compartment and then extruded through the membranes by ABC transporter and export systems (Figure 3). PSA confers a selective advantage to bacteria in the human nervous system and is associated with meningitis or other neurological conditions. Bacteria may also have PSA with α2–9 linkages or alternating α2–8 and α2–9 linkages.

In mammals, PSTs synthesize PSA by the addition of individual sialic acid residues in a processive fashion. Like the other sialyltransferases, mammalian PSTs are inverting enzymes of the GT29 family (Table 3). In addition to four sialyl motifs, ST8SIAII and ST8SIAIV (formerly STX and PST, respectively) have a unique, conserved, polybasic PST domain (PSTD motif) (Table 5) (67), which is absent from the other types of sialyltransferases. Basic residues in the PSTD motif are responsible for acceptor substrate recognition (126, 127).

In bacteria, the PSA capsule is synthesized by GT38 family PST that are inverting enzymes. In *E. coli*, PST has only 5.4% sequence identity with the human enzyme (128). The human PST equivalent from *Nm* has <10% sequence identity with human PST and has a requirement for Mg^2+^ (129). Kinetic experiments of His and Pro mutants of the PST from *Nm* suggested that the HP motif contributes to CMP-sialic acid but not acceptor binding. The acceptors can be a glycolipid containing two sialic acid residues as a primer. Gal-terminating glycans of glycopeptides, including the T antigen linked to Ser, also served as acceptor substrates for the PST form *Nm*. Different PSTs synthesize either the α2–8 or α2–9 linked polymers of bacterial PSA capsules.

## Methods to Study Glycosyltransferase Protein Structures and Functions

It is often difficult to produce sufficient pure enzyme in order to analyze protein structure by X-ray crystallography. In addition, the protein may not show exactly the same properties in a crystal, compared to solution and body fluids. To approximate the protein structure present in the natural environment, protein NMR studies have been helpful (130). Enzyme substrate or inhibitor interactions have been determined by biochemical kinetics studies but can also be studied by MS and Saturation Transfer Difference (STD) NMR (131, 132). Conformational dynamics of proteins to understand molecular recognition can be achieved by molecular dynamics simulation and docking programs, which requires knowledge of protein structure. Theoretical modeling has been undertaken to predict protein structure, substrate binding, and dynamic properties of GTs. Thus, the three-dimensional interactions between substrates and enzyme protein, cofactor binding sites, ligand flexibility, and movements can be estimated by computational methods (133, 134). Multivariate data analysis of the amino acid property patterns also helps to predict a protein fold (135).

New enzymes can be designed based on knowledge of protein structure and substrate binding. For example, the blood group B GTB Gal-transferase has been re-designed with a model Epimer Propensity Index (EPI) to transfer Glc instead of Gal (136). The orientation of the sugar donor in the folded enzyme is highly conserved. The R228K mutant of β4GalT1 has higher Glc-transferase activity due to the inability to effectively bind the axial 4-hydroxyl of Gal (23). Similarly, GTB modeling correctly predicted a higher Glc-transferase activity of GTB in the presence of the unnatural UDP-Glc donor upon increasing the sizes of Ser185 to Asn and Cys (136).

One approach to developing good GT inhibitors is to obtain qualitative and quantitative information on the substrate binding sites from NMR spectroscopy. STD NMR measures the signals of the unbound substrate, which is then compared to those of the bound substrate. Saturation transferred from the enzyme to specific sites of the bound substrate is seen as an attenuation of resonance signals. The difference spectra at different ligand concentrations allow to identify the bound substrate and to determine the binding affinity. In spin-lock filtering experiments, transverse relaxation of substrate signals is recorded, which is enhanced when the ligand is bound. Thus, signals are attenuated upon ligand binding. This process can be enhanced by using spin labels. The conformations and relative placements of bound GnT V substrates have been determined using transferred NOE and STD measurements (137).

In addition to these NMR experiments, surface plasmon resonsance (Bioacore) experiments can be used to determine the binding affinities (132). The ligand binding ability of GTs with and without donor or a number of potential inhibitors can be assessed with biotinylated substrates bound to a streptavidin-coated chip. The binding of donor and acceptor analogs to the blood group B enzyme GTB has also been analyzed by ITC combined with STD NMR titration (138). The results show the binding stoichiometry and binding affinity of one donor and acceptor molecule per protein, the thermodynamics, enthalpy, and entropy changes upon binding as well as the dissociation constants. The study also emphasizes that there can be differences in binding substrate analogs that should be considered.

Electrospray-mass spectrometry (ES-MS) has been used to determine the thermodynamics and affinities of substrates (139). Association constants were measured from the relative abundance of ions in the EI-MS spectra for GTA and GTB in aqueous solution with native donor and acceptor substrates as well as substrate analogs, products, and metal ion cofactor. To confirm the retaining mechanism of the enzymes, a mutant of blood group A (GTA) GalNAc-transferase in solution containing UDP-GalNAc and Mn^2+^ was studied, as well as the similar mutant of GTB. The catalytic Glu303 was replaced with Cys. After Trypsin digestion, a covalent intermediate could be trapped. Thus, tandem MS using collision-induced dissociation confirmed that Cys303 in both GTA and GTB enzymes was responsible for forming the glycosyl-enzyme intermediate. The formation of trisaccharide product can also be proven by MS (68). Thus, the double displacement mechanism of these retaining GTs was supported by MS.

## Glycosyltransferase Inhibitors

Detailed knowledge of GT structure and function is the basis for the development of effective GT inhibitors that may re-direct glycan biosynthesis. GT substrates usually bind through a small number of essential hydrogen bonds or hydrophobic interactions. Thus, not all of the substituents of the donor sugar, the base of the nucleotide, or the sugars of the acceptor are critically involved in binding (140). Therefore, modifications of these residues can result in competitive inhibitors that still bind in the catalytic site but do not support catalysis. Inhibitors can be ligands that bind well to the enzyme but cannot be released easily, or interfere with catalysis either as donor substrate analogs, acceptor substrate analogs, transition state analogs, compounds that prevent conformational changes necessary for catalysis, or compounds that distort protein conformation (74, 77, 103). Small structural modifications of compounds can have a dramatic effect on their inhibitory activity. Inhibitors have been designed that interfere with conformational changes and flexible loop movements that are essential events for substrate binding and catalysis (141). Sugar donor analogs for ABO transferases (GTA and GTB), carrying a substituent at the uracil moiety, block the stacking of amino acids required for the proper folding of the internal loop. A heterocyclic compound inhibited GTB by interfering with its ability to bind metal ion, as well as donor and acceptor substrates. The compound does not appear to be structurally related to the acceptor but partly binds in the acceptor-binding site (142). A combination of crystal structure, Biacore, STD NMR, and docking experiments suggested that the inhibitor competes with binding of Fuc of the acceptor and the Mn^2+^ ion. Non-competitive inhibitors have also been described that potentially alter the structure of the enzyme leading to inactive proteins (77, 78). Modified nucleotide sugars are often recognized by GTs leading to transfer of unnatural sugars (143). Fluorescent groups modifying the base of the sugar-nucleotide can be useful as indicators of binding (144).

## Chemoenzymatic Synthesis of Shared Epitopes

The preparation of bacterial GTs that lack a transmembrane domain is relatively inexpensive and they can be used in chemoenzymatic synthesis not only of bacterial glycoconjugates but also for mammalian oligosaccharides and glycoproteins with specific epitopes (Table 1). Due to the variety of bacterial enzymes with different specificities, a diverse range of glycan structures can be synthesized and processed for use as vaccine, to prepare antibodies for passive immunity, and for further studies of glycan functions. Examples include the synthesis of the complete blood group Forssman antigen GalNAcα3GalNAcβ3Galα4Galβ4Glc-p-nitrophenyl by β3-GalNAc-transferase and α4-Gal-transferase from *Cj*, followed by α3-GalNAc-transferase from *Pm* (81, 140). The assembly of the entire blood group B determinant was achieved using GTs from *E. coli* O86 (145). Bacterial enzymes α4-Gal-transferase LgtC, β3-Gal(NAc)-transferase LgtD, and α2-Fuc-transferase WbsJ (146) efficiently synthesized the tumor-associated epitope Globo-H-hexasaccharide (Fucα2-Galβ1–3GalNAcβ1–3Gaαl–4Galβ1–4Glcβ-benzyl).

Knowing the amino acids and mechanisms involved in substrate binding and catalysis, bacterial enzymes or new mutant enzymes can be engineered for use in the production of new natural or unnatural glycan structures, or for more efficient synthesis of known structures (147). For example, new donor specificity can be engineered by mutating only one or two critical amino acids that convert the function of the enzyme (140, 148).

Phosphorylases can also reversibly form glycosidic linkages (149). They can have similarity to either inverting or retaining glycohydrolases or to GT-B-folded retaining GT (CAZy). Sugar-1-P can be used as a substrate for phosphorylases to produce a wealth of different glycans with regio-selectivity. An interesting combination of chemical and enzymatic synthesis of the T antigen and the Galβ1–3GlcNAc linkage has been achieved using a combination of galactokinase (GalK) from *E. coli* that synthesizes Gal-1-P, and a Galβ1–3 HexNAc phosphorylase from *Bifobacterium infantis* that has promiscuous acceptor specificity (150). These enzymes could add Gal in the presence of ATP to synthetic GalNAc- and GlcNAc- substrates with various aglycone groups. The phosphorylase has multiple DxD motifs and an Asp-rich domain at the C terminus. The T antigen was also synthesized from sucrose and GlcNAc using phosphorylase from *Bifidobacterium longum* (151) together with sucrose phosphorylase, UDP-Glc-hexose-1-P uridyltransferase and UDP-Glc 4-epimerase.

Glycosidases catalyze reversible reactions and have also been used to form sugar linkages using high concentrations of reactants. Glycosidases act with an inverting or retaining mechanism, utilizing a catalytically active nucleophile in the active site such as Asp or Glu. Mutant glycohydrolases that lack the catalytic base as well as hydrolase activity can be used to efficiently transfer a sugar to an acceptor substrate and synthesize specific linkages (glycosynthases). For example, large N-glycan-type oligosaccharides can be transferred to the GlcNAc residue linked to Asn of glycoproteins by a mutant endo-glucosaminidase that normally cleaves the chitobiose and releases the N-glycan. Thus, engineered glycosidases can be stereo-selective and very useful in achieving high yields of complex glycans (152).

## Concluding Remarks

It is astounding that proteins can be so different in amino acid sequence and yet become similar specific and effective catalysts for the transfer of sugars to proteins, lipids, and sugars and only have two major protein folds. Many possibilities are there for binding of donor and acceptor substrates but the transfer only involves inversion or retention of the anomeric configuration of the sugar. Mechanisms common to eukaryotes and bacteria include a change in protein conformation upon nucleotide sugar binding facilitating acceptor binding, and the action of a base (Glu, Asp, His) that deprotonates the hydroxyl to be glycosylated, which then becomes a nucleophile that results in cleavage of the sugar from the donor substrate. Bacterial and mammalian enzymes are often comparable in their action so that mammalian epitopes can easily be synthesized with bacterial enzymes, for example, to produce vaccines for cancer. However, the bacterial world is much more complex, variable, and challenging. Knowledge of bacterial GTs can lead to the synthesis of glycans, enzyme substrates, and antigens to study their biological functions and role in disease and to synthesize vaccines against specific pathogenic strains of bacteria. Bacteria may have evolved to express the GTs that make human-like structures, giving them a selective advantage. Most of the time, bacteria and human beings are symbiotic or compatible but once in a while, the mimicry of bacteria can lead to infection and serious consequences. We speculate that bacterial and mammalian enzymes with similar functions may have evolved in parallel, or may be derived from an ancient common ancestor. There may have been exchange of genes between these species (horizontal gene transfer), or GTs may be derived by convergent evolution. The many similar genes of a particular family may have been derived by gene duplication from an ancestral gene.

Further detailed understanding of GT structures and mechanisms helps to visualize how amino acids cooperate in forming a catalytic site, predict their functions, and to gain valuable insight into the syntheses of complex glycans in mammals and in our close neighbors, bacteria. Both, the bacterial world and human beings can benefit from this relationship. In addition, inhibitors of bacterial GTs may help to eliminate virulence factors, and this is an urgently needed goal in light of growing antibiotic resistance.

## Conflict of Interest Statement

The author declares that the research was conducted in the absence of any commercial or financial relationships that could be construed as a potential conflict of interest.

## References

[1] Petit D Teppa RE Petit JM Harduin-Lepers A. A practical approach to reconstruct evolutionary history of animal sialyltransferases and gain insights into the sequence-function relationships of Golgi-glycosyltransferases. Methods Mol Biol (2013) 1022:73–97. 10.1007/978-1-62703-465-4_7 23765655

[2] Lombard V Golaconda Ramulu H Drula E Coutinho PM Henrissat B. The carbohydrate-active enzymes database (CAZy) in 2013. Nucleic Acids Res (2014) 42(Database issue):D490–5. 10.1093/nar/gkt1178 24270786 PMC3965031

[3] Baycin Hizal D Wolozny D Colao J Jacobson E Tian Y Krag SS Glycoproteomic and glycomic databases. Clin Proteomics (2014) 11:15. 10.1186/1559-0275-11-15 24725457 PMC3996109

[4] Yen TY Dutta SM Litsakos-Cheung C Corona AA Timpe LC Macher BA. Overcoming challenges and opening new opportunities in glycoproteomics. Biomolecules (2013) 3:270–86. 10.3390/biom3020270 24790834 PMC4002168

[5] Lundborg M Modhukur V Widmalm G. Glycosyltransferase functions of *E. coli* O-antigens. Glycobiology (2010) 20:366–8. 10.1093/glycob/cwp185 19926726

[6] Varki A. Biological roles of oligosaccharides: all of the theories are correct. Glycobiology (1993) 3:97–130. 10.1093/glycob/3.2.97 8490246 PMC7108619

[7] Aebi M. N-linked protein glycosylation in the ER. Biochim Biophys Acta (2013) 1833:2430–7. 10.1016/j.bbamcr.2013.04.001 23583305

[8] Brockhausen I. The role of galactosyltransferases in celi surface functions and in the immune system. Drug News Perspect (2006) 19:401–9. 10.1358/dnp.2006.tg.j.t02t4g1 17080203

[9] Taniguchi N Korekane H. Branched N-glycans and their implications for cell adhesion, signaling and clinical applications for cancer biomarkers and in therapeutics. BMB Rep (2011) 44:772–81. 10.5483/BMBRep.2011.44.12.772 22189679

[10] Boscher C Dennis JW Nabi IR. Glycosylation, galectins and cellular signaling. Curr Opin Cell Biol (2011) 23:383–92. 10.1016/j.ceb.2011.05.001 21616652

[11] Dalziel M Crispin M Scanlan CN Zitzmann N Dwek RA. Emerging principles for the therapeutic exploitation of glycosylation. Science (2014) 343:1235681. 10.1126/science.1235681 24385630

[12] Brockhausen I Schutzbach J Kuhns W. Glycoproteins and their relationship to human disease. Acta Anat (1998) 161:36–78. 10.1159/000046450 9780351

[13] Nothaft H Szymanski CM. Bacterial protein N-glycosylation: new perspectives and applications. J Biol Chem (2013) 288:6912–20. 10.1074/jbc.R112.417857 23329827 PMC3591601

[14] Brockhausen I. Pathways of O-glycan biosynthesis in cancer cells. Biochim Biophys Acta (1999) 1473:67–95. 10.1016/S0304-4165(99)00170-1 10580130

[15] Brockhausen I. Biosynthesis of complex mucin-type O-glycans. In: Mander L Lui H-W Wang PG editors. Comprehensive Natural Products II Chemistry and Biology: Carbohydrates, Nucleosides and Nucleic Acids. (Vol. 6), Oxford: Elsevier (2010). p. 315–50. 10.1016/B978-008045382-8.00643-2

[16] Tarp MA Clausen H. Mucin-type O-glycosylation and its potential use in drug and vaccine development. Biochim Biophys Acta (2008) 1780:546–63. 10.1016/j.bbagen.2007.09.010 17988798

[17] Fritz TA Hurley JH Trinh LB Shiloach J Tabak LA. The beginnings of mucin biosynthesis: the crystal structure of UDP-GalNAc: polypeptide alpha-N-acetylgalactosaminyltransferase-T1. Proc Natl Acad Sci U S A (2004) 101:15307–12. 10.1073/pnas.0405657101 15486088 PMC524453

[18] Fritz TA Raman J Tabak LA. Dynamic association between the catalytic and lectin domains of human UDP-GalNAc: polypeptide-N-acetylgalactosaminyltransferase-2. J Biol Chem (2006) 281:8613–9. 10.1074/jbc.M513590200 16434399

[19] Ünligil UM Rini JM. Glycosyltransferase structure and mechanism. Curr Opin Struct Biol (2000) 10:510–7. 10.1016/S0959-440X(00)00124-X 11042447

[20] Pak JE Arnoux P Zhou S Sivarajah P Satkunarajah M Xing X X-ray crystal structure of leukocyte type core 2 beta1,6-N-acetylglucosaminyltransferase. Evidence for a convergence of metal ion-independent glycosyltransferase mechanism. J Biol Chem (2006) 281:26693–701. 10.1074/jbc.M603534200 16829524

[21] Pak JE Satkunarajah M Seetharaman J Rini JM. Structural and mechanistic characterization of leukocyte-type core 2 β1,6-N-acetylglucosaminy ltransferase: a metal-ion-independent GT-A glycosyltransferase. J Mol Biol (2011) 414:798–811. 10.1016/j.jmb.2011.10.039 22056345

[22] Ramakrishnan B Balaji PV Qasba PK. Crystal structure of beta1,4-galactosyltransferase. complex with UDP-gal reveals an oligosaccharide acceptor binding site. J Mol Biol (2002) 318:491–502. 10.1016/S0022-2836(02)00020-7 12051854

[23] Ramakrishnan B Boeggeman E Qasba PK. Mutation of arginine 228 to lysine enhances the glucosyltransferase activity of bovine beta-1,4-galactosyl transferase I. Biochemistry (2005) 44:3202–10. 10.1021/bi0479454 15736931

[24] Tsutsui Y Ramakrishnan B Qasba PK. Crystal structures of β-1,4-galactosyltransferase 7 enzyme reveal conformational changes and substrate binding. J Biol Chem (2013) 288:31963–70. 10.1074/jbc.M113.509984 24052259 PMC3814792

[25] Sun HY Lin SW Ko TP Pan JF Liu CL Lin C Structure and mechanism of *Helicobacter pylori* fucosyltransferase. A basis for lipopolysaccharide variation and inhibitor design. J Biol Chem (2007) 282:9973–82. 10.1074/jbc.M610285200 17251184

[26] Ihara H Ikeda Y Toma S Wang X Suzuki T Gu J Crystal structure of mammalian alpha1,6-fucosyltransferase, FUT8. Glycobiology (2007) 17:455–66. 10.1093/glycob/cwl079 17172260

[27] Brzezinski K Stepkowski T Panjikar S Bujacz G Jaskolski M. High-resolution structure of NodZ fucosyltransferase involved in the biosynthesis of the nodulation factor. Acta Biochim Pol (2007) 54:537–49. 17762900

[28] Rakic B Rao FV Freimann K Wakarchuk W Strynadka NCJ Withers SG. Structure-based mutagenic analysis of mechanism and substrate specificity in mammalian glycosyltransferases: porcine ST3Gal-I. Glycobiology (2013) 23:536–45. 10.1093/glycob/cwt001 23300007

[29] Iwatani T Okino N Sakakura M Kajiwara H Takakura Y Kimura M Crystal structure of alpha/beta-galactoside alpha2,3-sialyltransferase from a luminous marine bacterium, *Photobacterium phosphoreum*. FEBS Lett (2009) 583:2083–7. 10.1016/j.febslet.2009.05.032 19467231

[30] Chiu CPC Lairson LL Gilbert M Wakarchuk WW Withers SG Strynadka NCJ. Structural analysis of the R-2,3-sialyltransferase Cst-I from *Campylobacter jejuni* in Apo and substrate-analogue bound forms. Biochemistry (2007) 46:7196–204. 10.1021/bi602543d 17518445

[31] Lin LYC Rakic B Chiu CPC Lameignere E Wakarchuk WW Withers SG Structure and mechanism of the lipooligosaccharide sialyltransferase from *Neisseria meningitidis*. J Biol Chem (2011) 286:37237–48. 10.1074/jbc.M111.249920 21880735 PMC3199471

[32] Meng L Forouhar F Thieker D Gao Z Ramiah A Moniz H Enzymatic basis for N-glycan sialylation. Structure of rat alpha2,6-sialyltransferase (st6gal1) reveals conserved and unique features for glycan sialylation. J Biol Chem (2013) 288:34680–98. 10.1074/jbc.M113.519041 24155237 PMC3843080

[33] Kuhn B Benz J Greif M Engel AM Sobek H Rudolph MG. The structure of human alpha-2,6-sialyltransferase reveals the binding mode of complex glycans. Acta Crystallogr D Biol Crystallogr (2013) D69:1826–38. 10.1107/S0907444913015412 23999306

[34] Ni L Chokhawala HA Cao H Henning R Ng L Huang S Crystal structures of *Pasteurella multocida* sialyltransferase complexes with acceptor and donor analogues reveal substrate binding sites and catalytic mechanism. Biochemistry (2007) 46:6288–98. 10.1021/bi700346w 17487984

[35] Kakuta Y Okino N Kajiwara H Ichikawa M Takakura Y Ito M Crystal structure of *Vibrionaceae Photobacterium* sp. JT-ISH-224 α2,6-sialyltransferase in a ternary complex with donor product CMP and acceptor substrate lactose: catalytic mechanism and substrate recognition. Glycobiology (2008) 18:66–73. 10.1093/glycob/cwm119 17962295

[36] Lee HJ Lairson LL Rich JR Lameignere E Wakarchuk WW Withers SG Structural and kinetic analysis of substrate binding to the sialyltransferase Cst-II from *Campylobacter jejuni*. J Biol Chem (2011) 286:35922–32. 10.1074/jbc.M111.261172 21832050 PMC3195623

[37] Kim DU Yoo JH Lee YJ Kim KS Cho HS. Structural analysis of sialyltransferase PM0188 from *Pasteurella multocida* complexed with donor analogue and acceptor sugar. BMB Rep (2008) 41:48–54. 10.5483/BMBRep.2008.41.1.048 18304450

[38] Kubota T Shiba T Sugioka S Furukawa S Sawaki H Kato R Structural basis of carbohydrate transfer activity by human UDP-GalNAc: polypeptide alpha-N-acetylgalactosaminyltransferase (pp-GalNAc-T10). J Mol Biol (2006) 359:708–27. 10.1016/j.jmb.2006.03.061 16650853

[39] Patenaude SI Seto NO Borisova SN Szpacenko A Marcus SL Palcic MM The structural basis for specificity in human ABO(H) blood group biosynthesis. Nat Struct Biol (2002) 9:685–90. 10.1038/nsb832 12198488

[40] Yazer MH Palcic MM. The importance of disordered loops in ABO glycosyltransferases. Transfus Med Rev (2005) 19:210–6. 10.1016/j.tmrv.2005.02.008 16010651

[41] Gastinel LN Bignon C Misra AK Hindsgaul O Shaper JH Joziasse DH. Bovine alpha1,3-galactosyltransferase catalytic domain structure and its relationship with ABO histo-blood group and glycosphingolipid glycosyltransferases. EMBO J (2001) 20:638–49. 10.1093/emboj/20.4.638 11179209 PMC145412

[42] Boix E Zhang Y Swaminathan GJ Brew K Acharya KR. Structural basis of ordered binding of donor and acceptor substrates to the retaining glycosyltransferase, alpha-1,3-galactosyltransferase. J Biol Chem (2002) 277:28310–8. 10.1074/jbc.M202631200 12011052

[43] Alfaro JA Zheng RB Persson M Letts JA Polakowski R Bai Y ABO(H) blood group A and B glycosyltransferases recognize substrate via specific conformational changes. J Biol Chem (2008) 283:10097–108. 10.1074/jbc.M708669200 18192272

[44] Thiyagarajan N Pham TTK Stinson B Sundriyal A Tumbale P Lizotte-Waniewski M Structure of a metal-independent bacterial glycosyltransferase that catalyzes the synthesis of histo-blood group A antigen. Sci Rep (2012) 2:940. 10.1038/srep00940 23230506 PMC3516806

[45] Persson K Ly HD Dieckelmann M Wakarchuk WW Withers SG Strynadka NCJ. Crystal structure of the retaining galactosyltransferase LgtC from *Neisseria meningitidis* in complex with donor and acceptor sugar analogs. Nat Struct Biol (2001) 8:166–75. 10.1038/84168 11175908

[46] Musumeci MA Hug I Scott NE Ielmini MV Foster LJ Wang PG In vitro activity of *Neisseria meningitidis* PglL O-oligosaccharyltransferase with diverse synthetic lipid donors and a UDP-activated sugar. J Biol Chem (2013) 288:10578–87. 10.1074/jbc.M112.432815 23460642 PMC3624439

[47] Musumeci MA Faridmoayer A Watanabe Y Feldman MF. Evaluating the role of conserved amino acids in bacterial O-oligosaccharyltransferases by in vivo, in vitro and limited proteolysis assays. Glycobiology (2014) 24:39–50. 10.1093/glycob/cwt087 24092836

[48] Whitfield C Trent MS. Biosynthesis and export of bacterial lipopolysaccharides. Annu Rev Biochem (2014) 83:99–128. 10.1146/annurev-biochem-060713-035600 24580642

[49] Rush JS Alaimo C Robbiani R Wacker M Waechter CJ. A novel epimerase that converts GlcNAc-P-P-undecaprenol to GalNAc-P-P-undecaprenol in *Escherichia coli* O157. J Biol Chem (2010) 285:1671–80. 10.1074/jbc.M109.061630 19923219 PMC2804325

[50] Freinkman E Chng SS Kahne D. The complex that inserts lipopolysaccharide into the bacterial outer membrane forms a two-protein plug-and-barrel. Proc Natl Acad Sci USA (2011) 108:2486–91. 10.1073/pnas.1015617108 21257904 PMC3038725

[51] Greenfield LK Whitfield C. Synthesis of lipopolysaccharide O-antigens by ABC transporter-dependent pathways. Carbohydr Res (2012) 356:12–24. 10.1016/j.carres.2012.02.027 22475157

[52] Coutinho PM Deleury E Davies GJ Henrissat B. An evolving hierarchical family classification for glycosyltransferases. J Mol Biol (2003) 328:307–17. 10.1016/S0022-2836(03)00307-3 12691742

[53] Li T Simonds L Kovrigin EL Noel KD. In vitro biosynthesis and chemical identification of UDP-N-acetyl-d-quinovosamine (UDP-d-QuiNAc). J Biol Chem (2014) 289:18110–20. 10.1074/jbc.M114.555862 24817117 PMC4140256

[54] Wang S Tanaka H Hindsgaul O Lam JS Brockhausen I. A convenient synthesis of GDP-d-rhamnose: the donor substrate for d-rhamnosyltransferase WbpZ from *Pseudomonas aeruginosa* PAO1. Bioorg Med Chem Lett (2013) 23:3491–5. 10.1016/j.bmcl.2013.04.051 23664878

[55] Montoya-Peleaz PJ Riley JG Szarek WA Valvano MA Schutzbach JS Brockhausen I. Identification of a UDP-Gal: GlcNAc-R galactosyltransferase activity in *Escherichia coli* VW187. Bioorg Med Chem Lett (2005) 15:1205–11. 10.1016/j.bmcl.2004.11.077 15686943

[56] Albesa-Jové D Giganti D Jackson M MAlzari P Guerin ME. Structure–function relationships of membrane-associated GT-B glycosyltransferases. Glycobiology (2014) 24:108–24. 10.1093/glycob/cwt101 24253765 PMC3907083

[57] Breton C Fournel-Gigleux S Palcic MM. Recent structures, evolution and mechanisms of glycosyltransferases. Curr Opin Struct Biol (2012) 22:540–9. 10.1016/j.sbi.2012.06.007 22819665

[58] Lairson LL Henrissat B Davies GJ Withers SG. Glycosyltransferases: structures, functions, and mechanisms. Annu Rev Biochem (2008) 77:521–55. 10.1146/annurev.biochem.76.061005.092322 18518825

[59] Namdjou DJ Chen HM Vinogradov E Brochu D Withers SG Wakarchuk WW. A beta-1,4-galactosyltransferase from *Helicobacter pylori* is an efficient and versatile biocatalyst displaying a novel activity for thioglycoside synthesis. Chembiochem (2008) 9:1632–40. 10.1002/cbic.200700775 18491328

[60] Breton C Oriol R Imberty A. Conserved structural features in eukaryotic and prokaryotic fucosyltransferases. Glycobiology (1998) 8:87–94. 10.1093/glycob/8.1.87 9451017

[61] Narimatsu H. Construction of a human glycogene library and comprehensive functional analysis. Glycoconj J (2004) 21:17–24. 10.1023/B:GLYC.0000043742.99482.01 15467393

[62] Oriol R Mollicone R Cailleau A Balanzino L Breton C. Divergent evolution of fucosyltransferase genes from vertebrates, invertebrates, and bacteria. Glycobiology (1999) 9:323–34. 10.1093/glycob/9.4.323 10089206

[63] Martinez-Duncker I Mollicone R Candelier JJ Breton C Oriol R. A new superfamily of protein-O-fucosyltransferases, alpha2-fucosyltransferases, and alpha6-fucosyltransferases: phylogeny and identification of conserved peptide motifs. Glycobiology (2003) 13:1C–5C. 10.1093/glycob/cwg113 12966037

[64] Li M Liu XW Shao J Shen J Jia Q Yi W Characterization of a novel alpha1,2-fucosyltransferase of *Escherichia coli* O128: b12 and functional investigation of its common motif. Biochemistry (2008) 47:378–87. 10.1021/bi701345v 18078329

[65] Patel RY Balaji PV. Identification of linkage-specific sequence motifs in sialyltransferases. Glycobiology (2006) 16:108–16. 10.1093/glycob/cwj046 16207893

[66] Freiberger F Claus H Gunzel A Oltmann-Norden I Vionnet J Muhlenhoff M Biochemical characterization of a *Neisseria meningitidis* polysialyltransferase reveals novel functional motifs in bacterial sialyltransferases. Mol Microbiol (2007) 65:1258–75. 10.1111/j.1365-2958.2007.05862.x 17662040 PMC2169525

[67] Nakata D Zhang L Troy IIFA. Molecular basis for polysialylation: a novel polybasic polysialyltransferase domain (PSTD) of 32 amino acids unique to the α2,8-polysialyltransferases is essential for polysialylation. Glycoconj J (2006) 23:423–36. 10.1007/s10719-006-6356-5 16897183

[68] Soya N Fang Y Palcic MM Klassen JS. Trapping and characterization of covalent intermediates of mutant retaining glycosyltransferases. Glycobiology (2011) 21:547–52. 10.1093/glycob/cwq190 21098513

[69] Amado M Almeida R Schwientek T Clausen H. Identification and characterization of large galactosyltransferase gene families: galactosyltransferases for all functions. Biochim Biophys Acta (1999) 1473:35–53. 10.1016/S0304-4165(99)00168-3 10580128

[70] Ramakrishnan B Boeggeman E Qasba PK. Effect of the Met344His mutation on the conformational dynamics of bovine beta-1,4-galactosyltransferase: crystal structure of the Met344His mutant in complex with chitobiose. Biochemistry (2004) 43:12513–22. 10.1021/bi049007 15449940

[71] Ramasamy V Ramakrishnan B Boeggeman E Ratner DM Seeberger PH Qasba PK. Oligosaccharide preferences of beta1,4-galactosyltransferase-I: crystal structures of Met340His mutant of human beta1,4-galactosyltransferase-I with a pentasaccharide and trisaccharides of the N-glycan moiety. J Mol Biol (2005) 353:53–67. 10.1016/j.jmb.2005.07.050 16157350

[72] Qasba PK Ramakrishnan B Boeggeman E. Structure and function of β-1,4-galactosyltransferase. Curr Drug Targets (2008) 9:292–309. 10.2174/138945008783954943 18393823 PMC2365515

[73] Xu C Liu B Hu B Han Y Feng L Allingham J Biochemical characterization of UDP-Gal: GlcNAc-pyrophosphate-lipid beta1,4-galactosyltransferase WfeD, a new enzyme from *Shigella boydii* type 14 that catalyzes the second step in O-antigen repeating-unit synthesis. J Bacteriol (2011) 193:449–59. 10.1128/JB.00737-10 21057010 PMC3019819

[74] Gao Y Lazar C Szarek WA Brockhausen I. Specificity of beta4galactosyltr ansferase inhibitor 2-naphthyl 2-butanamido-2-deoxy-1-thio-beta-d-glucopy ranoside. Glycoconj J (2010) 27:673–84. 10.1007/s10719-010-9312-3 20976621

[75] Riley JG Menggad M Montoya-Peleaz P Szarek WA Marolda CL Valvano MA The wbbD gene of *E. coli* strain VW187 (O7: K1) encodes a UDP-Gal: GlcNAc(alpha)-pyrophosphate-R (beta)1,3-galactosyltransferase involved in the biosynthesis of O7-specific lipopolysaccharide. Glycobiology (2005) 15:605–13. 10.1093/glycob/cwi038 15625181

[76] Ju T Cummings RD. A unique molecular chaperone Cosmc required for activity of the mammalian core 1 beta3-galactosyltransferase. Proc Natl Acad Sci U S A (2002) 99:16613–8. 10.1073/pnas.262438199 12464682 PMC139192

[77] Gao Y Aryal RP Ju T Cummings RD Gahlay G Jarvis DL Acceptor specificities and selective inhibition of recombinant human Gal- and GlcNAc-transferases that synthesize O-glycan core structures 1, 2, 3 and 4 of O-glycans. Biochim Biophys Acta (2013) 1830:4274–81. 10.1016/j.bbagen.2013.04.001 23578692 PMC4002258

[78] Wang S Czuchry D Liu B Vinnikova AN Gao Y Vlahakis JZ Characterization of Two UDP-Gal: GalNAc-diphosphate-lipid β1,3-galactosyltransferases WbwC from *Escherichia coli* serotypes O104 and O5. J Bacteriol (2014) 196(17):3122–33. 10.1128/JB.01698-14 24957618 PMC4135647

[79] Steffensen R Carlier K Wiels J Levery SB Stroud M Cedergren B Cloning and expression of the histo-blood group Pk UDP-galactose: Ga1beta-4G1cbeta1-cer alpha1, 4-galactosyltransferase. Molecular genetic basis of the p phenotype. J Biol Chem (2000) 275:16723–9. 10.1074/jbc.M000728200 10747952

[80] Suzuki N Yamamoto K. Molecular cloning of pigeon UDP-galactose: beta-d-galactoside alpha1,4-galactosyltransferase and UDP-galactose: beta-d-galactoside beta1,4-galactosyltransferase, two novel enzymes catalyzing the formation of Gal alpha1-4Gal beta1-4Gal beta1-4GlcNAc sequence. J Biol Chem (2010) 285:5178–87. 10.1074/jbc.M109.018663 19959475 PMC2820745

[81] Houliston RS Bernatchez S Karwaski MF Mandrell RE Jarrell HC Wakarchuk WW Complete chemoenzymatic synthesis of the Forssman antigen using novel glycosyltransferases identified in *Campylobacter jejuni* and *Pasteurella multocida*. Glycobiology (2009) 19:153–9. 10.1093/glycob/cwn117 18955372

[82] Chan PH Cheung AH Okon M Chen HM Withers SG McIntosh LP. Investigating the structural dynamics of α-1,4-galactosyltransferase C from *Neisseria meningitidis* by nuclear magnetic resonance spectroscopy. Biochemistry (2013) 52:320–32. 10.1021/bi301317d 23259770

[83] Yi W Perali RS Eguchi H Motari E Woodward R Wang PG. Characterization of a bacterial beta1,3-galactosyltransferase with application in the synthesis of tumor-associated t-antigen mimics. Biochemistry (2008) 47:1241–8. 10.1021/bi7020712 18179256

[84] Brockhausen I Hu B Liu B Lau K Szarek WA Wang L Characterization of two β1,3-glucosyltransferases from the *Escherichia coli* serotypes O56 and O152. J Bacteriol (2008) 190:4922–32. 10.1128/JB.00160-08 18487334 PMC2446995

[85] Gao Y Liu B Strum S Schutzbach J Druzhinina TN Utkina NS Biochemical characterization of WbdN, a beta1,3-glucosyltransferase involved in O-antigen synthesis in enterohemorrhagic *Escherichia coli* O157. Glycobiology (2012) 22:1092–102. 10.1093/glycob/cws081 22556057

[86] Bernatchez S Gilbert M Blanchard MC Karwaski MF Li J DeFrees S Variants of the β1,3-galactosyltransferase CgtB from the bacterium *Campylobacter jejuni* have distinct acceptor specificities. Glycobiology (2007) 17:1333–43. 10.1093/glycob/cwm090 17766267

[87] Liu XW Xia C Li L Guan WY Pettit N Zhang HC Characterization and synthetic application of a novel beta1,3-galactosyltransferase from *Escherichia coli* O55: H7. Bioorg Med Chem (2009) 17:4910–5. 10.1016/j.bmc.2009.06.005 19560364

[88] Seko A Yamashita K. Characterization of a novel galactose beta1,3-N-acetylglucosaminyltransferase (beta3Gn-T8): the complex formation of beta3Gn-T2 and beta3Gn-T8 enhances enzymatic activity. Glycobiology (2005) 15:943–51. 10.1093/glycob/cwi082 15917431

[89] Togayachi A Kozono Y Kuno A Ohkura T Sato T Hirabayashi J Beta3GnT2 (B3GNT2), a major polylactosamine synthase: analysis of B3GNT2-deficient mice. Methods Enzymol (2010) 479:185–204. 10.1016/S0076-6879(10)79011-X 20816167

[90] Yang X Qin W Lehotay M Toki D Dennis P Schutzbach JS Soluble human core 2 beta6-N-acetylglucosaminyltransferase C2GnT1 requires its conserved cysteine residues for full activity. Biochim Biophys Acta (2003) 1648:62–74. 10.1016/S1570-9639(03)00105-5 12758148

[91] Toki D Sarkar M Yip B Reck F Joziasse D Fukuda M Expression of stable human O-glycan core 2 beta-1,6-N-acetylglucosaminyltransferase in Sf9 insect cells. Biochem J (1997) 325:63–9. 9224630 10.1042/bj3250063 PMC1218529

[92] Peng W Pranskevich J Nycholat C Gilbert M Wakarchuk W Paulson JC *Helicobacter pylori* β1,3-N-acetylglucosaminyltransferase for versatile synthesis of type 1 and type 2 poly-LacNAcs on N-linked, O-linked and I-antigen glycans. Glycobiology (2012) 22:1453–64. 10.1093/glycob/cws101 22786570 PMC3481905

[93] Guan W Ban L Cai L Li L Chen W Liu X Combining carbochips and mass spectrometry to study the donor specificity for the *Neisseria meningitidis* β1,3-N-acetylglucosaminyltransferase LgtA. Bioorg Med Chem Lett (2011) 21:5025–8. 10.1016/j.bmcl.2011.04.100 21704524

[94] Wang G Boulton PG Chan NW Palcic MM Taylor DE. Novel *Helicobacter pylori* alpha1,2-fucosyltransferase, a key enzyme in the synthesis of Lewis antigens. Microbiology (1999) 145(Pt 11):3245–53. 10589734 10.1099/00221287-145-11-3245

[95] Li M Shen J Liu X Shao J Yi W Chow CS Identification of a new alpha1,2-fucosyltransferase involved in O-antigen biosynthesis of *Escherichia coli* O86: B7 and formation of H-type 3 blood group antigen. Biochemistry (2008) 47:11590–7. 10.1021/bi801067s 18842005

[96] Pettit N Styslingera T Meia Z Hana W Zhaoa G Wang PG. Characterization of WbiQ: an α1,2-fucosyltransferase from *Escherichia coli* O127: K63(B8), and synthesis of H-type 3 blood group antigen. Biochem Biophys Res Commun (2010) 402:190–5. 10.1016/j.bbrc.2010.08.087 20801103 PMC3441828

[97] Sperandio M Gleissner CA Ley K. Glycosylation in immune cell trafficking. Immunol Rev (2009) 230:97–113. 10.1111/j.1600-065X.2009.00795.x 19594631 PMC2745114

[98] Mollicone R Moore SE Bovin N Garcia-Rosasco M Candelier JJ Martinez-Duncker I Activity, splice variants, conserved peptide motifs, and phylogeny of two new alpha1,3-fucosyltransferase families (FUT10 and FUT11). J Biol Chem (2009) 284:4723–38. 10.1074/jbc.M809312200 19088067

[99] Dupuy F Germot A Julien R Maftah A. Structure/function study of Lewis alpha3- and alpha3/4-fucosyltransferases: the alpha1,4 fucosylation requires an aromatic residue in the acceptor-binding domain. Glycobiology (2004) 14:347–56. 10.1093/glycob/cwh053 14718375

[100] Ma B Lau LH Palcic MM Hazes B Taylor DEA. Single aromatic amino acid at the carboxyl terminus of *Helicobacter pylori* alpha1,3/4 fucosyltransferase determines substrate specificity. J Biol Chem (2005) 280:36848–56. 10.1074/jbc.M504415200 16150700

[101] Lin SW Yuan TM Li JR Lin CH. Carboxyl terminus of *Helicobacter pylori* alpha1,3-fucosyltransferase determines the structure and stability. Biochemistry (2006) 45:8108–16. 10.1021/bi0601297 16800635

[102] Zhang L Lau K Cheng J Yu H Li Y Sugiarto G *Helicobacter hepaticus* Hh0072 gene encodes a novel α1-3-fucosyltransferase belonging to CAZy GT11 family. Glycobiology (2010) 20:1077–88. 10.1093/glycob/cwq068 20466652 PMC2948817

[103] Bastida A Fernandez-Mayoralas A Arrayas RG Iradier F Carretero JC García-Junceda E. Heterologous over-expression of alpha-1,6-fucosyltransferase from *Rhizobium* sp.: application to the synthesis of the trisaccharide beta-d-GlcNAc(1-4)-[alpha-l-Fuc-(1-6)]-d-GlcNAc, study of the acceptor specificity and evaluation of polyhydroxylated indolizidines as inhibitors. Chemistry (2001) 7:2390–7. 10.1002/1521-3765(20010601)7:11<2390::AID-CHEM23900>3.0.CO;2-0 11446641

[104] Brew K Tumbale P Acharya KR. Family 6 glycosyltransferases in vertebrates and bacteria: inactivation and horizontal gene transfer may enhance mutualism between vertebrates and bacteria. J Biol Chem (2010) 285:37121–7. 10.1074/jbc.R110.176248 20870714 PMC2988317

[105] Yi W Shen J Zhou G Li J Wang PG. Bacterial homologue of human blood group A transferase. J Am Chem Soc (2008) 130:14420–1. 10.1021/ja805844y 18842049

[106] Jamaluddin H Tumbale P Ferns TA Thiyagarajan N Brew K Acharya KR. Crystal structure of alpha-1,3-galactosyltransferase (alpha3GT) in a complex with p-nitrophenyl-beta-galactoside (pNPbGal). Biochem Biophys Res Commun (2009) 385:601–4. 10.1016/j.bbrc.2009.05.111 19486884

[107] Harduin-Lepers A Mollicone R Delannoy P Oriol R. The animal sialyltransferases and sialyltransferase-related genes: a phylogenetic approach. Glycobiology (2005) 15:805–17. 10.1093/glycob/cwi063 15843597

[108] Troy FA. Polysialylation: from bacteria to brains. Glycobiology (1992) 2:5–23. 10.1093/glycob/2.1.5 1550990

[109] Amith SR Jayanth P Franchuk S Finlay T Seyrantepe V Beyaert R Neu1 desialylation of sialyl alpha-2,3-linked beta-galactosyl residues of TOLL-like receptor 4 is essential for receptor activation and cellular signaling. Cell Signal (2010) 22:314–24. 10.1016/j.cellsig.2009.09.038 19796680

[110] Burchell J Poulsom R Hanby A Whitehouse C Cooper L Clausen H An alpha2,3 sialyltransferase (ST3Gal I) is elevated in primary breast carcinomas. Glycobiology (1999) 9:1307–11. 10.1093/glycob/9.12.1307 10561455

[111] Picco G Julien S Brockhausen I Beatson R Antonopoulos A Haslam S Over-expression of ST3Gal-I promotes mammary tumorigenesis. Glycobiology (2010) 20:1241–50. 10.1093/glycob/cwq085 20534593 PMC2934706

[112] Whitehouse C Burchell J Gschmeissner S Brockhausen I Lloyd KO Taylor-Papadimitriou J. A transfected sialyltransferase that is elevated in breast cancer and localizes to the medial/trans-Golgi apparatus inhibits the development of core-2-based O-glycans. J Cell Biol (1997) 137:1229–41. 10.1083/jcb.137.6.1229 9182658 PMC2132526

[113] Dall’Olio F Malagolini N Serafini-Cessi F. The expression of soluble and cell-bound alpha 2,6 sialyltransferase in human colonic carcinoma CaCo-2 cells correlates with the degree of enterocytic differentiation. Biochem Biophys Res Commun (1992) 184:1405–10. 10.1016/S0006-291X(05)80039-7 1590800

[114] Taatjes DJ Roth J Weinstein J Paulson JC. Post-Golgi apparatus localization and regional expression of rat intestinal sialyltransferase detected by immunoelectron microscopy with polypeptide epitope-purified antibody. J Biol Chem (1988) 263:6302–9. 2452161

[115] Lehoux S Groux-Degroote S Cazet A Dhaenens CM Maurage CA Caillet-Boudin ML Transcriptional regulation of the human ST6GAL2 gene in cerebral cortex and neuronal cells. Glycoconj J (2010) 27:99–114. 10.1007/s10719-009-9260-y 19768537

[116] Houliston RS Vinogradov E Dzieciatkowska M Li J St. Michael F Karwaski MF Lipooligosaccharide of *Campylobacter jejuni*. Similarity with multiple types of mammalian glycans beyond gangliosides. J Biol Chem (2011) 286:12361–70. 10.1074/jbc.M110.181750 21257763 PMC3069439

[117] Yuki N Odaka M. Ganglioside mimicry as a cause of Guillain-Barré syn- drome. Curr Opin Neurol (2005) 18:557–61. 10.1097/01.wco.0000174604.42272.2d 16155440

[118] Wakerley BR Yuki N. Infectious and noninfectious triggers in Guillain-Barré syndrome. Expert Rev Clin Immunol (2013) 9:627–39. 10.1586/1744666X.2013.811119 23899233

[119] Chiu CPC Watts AG Lairson LL Gilbert M Lim D Wakarchuk WW Structural analysis of the sialyltransferase CstII from *Campylobacter jejuni* in complex with a substrate analog. Nat Struct Mol Biol (2004) 11:163–70. 10.1038/nsmb720 14730352

[120] Chan PHW Lairson LL Lee HJ Wakarchuk WW Strynadka NCJ Withers SG NMR spectroscopic characterization of the sialyltransferase CstII from *Campylobacter jejuni:* histidine 188 is the general base. Biochemistry (2009) 48:11220–30. 10.1021/bi901606n 19824695

[121] Schmölzer K Ribitsch D Czabany T Luley-Goedl C Kokot D Lyskowski A Characterization of a multifunctional α2,3-sialyltransferase from *Pasteurella dagmatis*. Glycobiology (2013) 23:1293–304. 10.1093/glycob/cwt066 23969291

[122] Drake PM Nathan JK Stock CM Chang PV Muench MO Nakata D Polysialic acid, a glycan with highly restricted expression, is found on human and murine leukocytes and modulates immune responses. J Immunol (2008) 181:6850–8. 10.4049/jimmunol.181.10.6850 18981104 PMC2718713

[123] Nakayama J Angata K Ong E Katsuyama T Fukuda M. Polysialic acid, a unique glycan that is developmentally regulated by two polysialyltransferases, PST and STX, in the central nervous system: from biosynthesis to function. Pathol Int (1998) 48:665–77. 10.1111/j.1440-1827.1998.tb03967.x 9778105

[124] Rollenhagen M Buettner FF Reismann M Jirmo AC Grove M Behrens GM Polysialic acid on neuropilin-2 is exclusively synthesized by the polysialyltransferase ST8SiaIV and attached to mucin-type o-glycans located between the b2 and c domain. J Biol Chem (2013) 288:22880–92. 10.1074/jbc.M113.463927 23801331 PMC3743467

[125] Cress BF Englaender JA He W Kasper D Linhardt RJ Koffas MA. Masquerading microbial pathogens: capsular polysaccharides mimic host-tissue molecules. FEMS Microbiol Rev (2014) 38:660–97. 10.1111/1574-6976.12056 24372337 PMC4120193

[126] Zapater JL Colley KJ. Sequences prior to conserved catalytic motifs of polysialyltransferase ST8Sia IV are required for substrate recognition. J Biol Chem (2012) 287:6441–53. 10.1074/jbc.M111.322024 22184126 PMC3307320

[127] Keys TG Fuchs HL Ehrit J Alves J Freiberger F Gerardy-Schahn R. Engineering the product profile of a polysialyltransferase. Nat Chem Biol (2014) 10:437–42. 10.1038/nchembio.1501.13 24727899

[128] Lindhout T Bainbridge CR Costain WJ Gilbert M Wakarchuk WW. Biochemical characterization of a polysialyltransferase from *Mannheimia haemolytica* A2 and comparison to other bacterial polysialyltransferases. PLoS ONE (2013) 8:e69888. 10.1371/journal.pone.0069888 23922842 PMC3724679

[129] Willis LM Gilbert M Karwaski MF Blanchard MC Wakarchuk WW. Characterization of the α-2,8-polysialyltransferase from *Neisseria meningitidis* with synthetic acceptors, and the development of a self-priming polysialyltransferase fusion enzyme. Glycobiology (2008) 18:177–86. 10.1093/glycob/cwm126 18000029

[130] Jayalakshmi V Biet T Peters T Krishna NR. Refinement of the conformation of UDP-galactose bound to galactosyltransferase using the STD NMR intensity-restrained CORCEMA optimization. J Am Chem Soc (2004) 126:8610–1. 10.1021/ja048703u 15250687

[131] Angulo J Langpap B Blume A Biet T Meyer B Rama Krishna N Blood group B galactosyltransferase: insights into substrate binding from NMR experiments. J Am Chem Soc (2006) 128:13529–38. 10.1021/ja063550r 17031966

[132] Rademacher C Landström J Sindhuwinata N Palcic MM Widmalm G Peters T. NMR-based exploration of the acceptor binding site of human blood group B galactosyltransferase with molecular fragments. Glycoconj J (2010) 27:349–58. 10.1007/s10719-010-9282-5 20217221

[133] Woods RJ Tessier MB. Computational glycoscience: characterizing the spatial and temporal properties of glycans and glycan–protein complexes. Curr Opin Struct Biol (2010) 20:575–83. 10.1016/j.sbi.2010.07.005 20708922 PMC3936461

[134] Patel RY Balaji PV. Fold-recognition and comparative modeling of human beta3GalT I, II, IV, V and VI and beta3GalNAcT I: prediction of residues conferring acceptor substrate specificity. J Mol Graph Model (2007) 26:255–68. 10.1016/j.jmgm.2006.12.003 17212986

[135] Rosén ML Edman M Sjöström M Wieslander A. Recognition of fold and sugar linkage for glycosyltransferases by multivariate sequence analysis. J Biol Chem (2004) 279:38683–92. 10.1074/jbc.M402925200 15148316

[136] Nakahara T Hindsgaul O Palcic MM Nishimura SI. Computational design and experimental evaluation of glycosyltransferase mutants: engineering of a blood type B galactosyltransferase with enhanced glucosyltransferase activity. Protein Eng Des Sel (2006) 19:571–8. 10.1093/protein/gzl046 17138593

[137] Macnaughtan MA Alvarez-Manilla MKG Venot A Glushka J Pierce JM Prestegard JH. NMR structural characterization of substrates bound to N-acetylglucosaminyltransferase V. J Mol Biol (2007) 366:1266–81. 10.1016/j.jmb.2006.12.015 17204285 PMC1808497

[138] Sindhuwinata N Grimm LL Weißbach S Zinn S Munoz E Palcic MM Thermodynamic signature of substrates and substrate analogs binding to human blood group B galactosyltransferase from isothermal titration calorimetry experiments. Biopolymers (2013) 99:784–95. 10.1002/bip.22297 23754468

[139] Soya N Shoemaker GK Palcic MM Klassen JS. Comparative study of substrate and product binding to the human ABO(H) blood group glycosyltransferases. Glycobiology (2009) 19:1224–34. 10.1093/glycob/cwp114 19648353

[140] Palcic MM. Glycosyltransferases as biocatalysts. Curr Opin Chem Biol (2011) 15:226–33. 10.1016/j.cbpa.2010.11.022 21334964

[141] Jørgensen R Pesnot T Lee HJ Palcic MM Wagner GK. Base-modified donor analogues reveal novel dynamic features of a glycosyltransferase. J Biol Chem (2013) 288:26201–8. 10.1074/jbc.M113.465963 23836908 PMC3764824

[142] Jørgensen R Grimm LL Sindhuwinata N Peters T Palcic MM. A glycosyltransferase inhibitor from a molecular fragment library simultaneously interferes with metal ion and substrate binding. Angew Chem Int Ed (2012) 51:4171–5. 10.1002/anie.201108345 22407594

[143] Schaefer K Albers J Sindhuwinata N Peters T Meyer BA. New concept for glycosyltransferase inhibitors: nonionic mimics of the nucleotide donor of the human blood group B galactosyltransferase. Chembiochem (2012) 13:443–50. 10.1002/cbic.201100642 22223604

[144] Pesnot T Palcic MM Wagner GK. A novel fluorescent probe for retaining galactosyltransferases. Chembiochem (2010) 11:1392–8. 10.1002/cbic.201000013 20533489

[145] Yi W Shao J Zhu L Li M Singh M Lu Y *Escherichia coli* O86 O-antigen biosynthetic gene cluster and stepwise enzymatic synthesis of human blood group B antigen tetrasaccharide. J Am Chem Soc (2005) 127:2040–1. 10.1021/ja045021y 15713070

[146] Su DM Eguchi H Yi W Li L Wang PG Xia C. Enzymatic synthesis of tumor-associated carbohydrate antigen Globo-H hexasaccharide. Org Lett (2008) 10:1009–12. 10.1021/ol703121h 18254640

[147] Chang A Singh S Phillips GN Jr. Thorson JS. Glycosyltransferase structural biology and its role in the design of catalysts for glycosylation. Curr Opin Biotechnol (2011) 22:800–8. 10.1016/j.copbio.2011.04.013 21592771 PMC3163058

[148] Hancock SM Vaughan MD Withers SG. Engineering of glycosidases and glycosyltransferases. Curr Opin Chem Biol (2006) 10:509–19. 10.1016/j.cbpa.2006.07.015 16905354

[149] Nakai H Kitaoka M Svensson B Ohtsubo K. Recent development of phosphorylases possessing large potential for oligosaccharide synthesis. Curr Opin Chem Biol (2013) 17:301–9. 10.1016/j.cbpa.2013.01.006 23403067

[150] Yu H Thon V Lau K Cai L Chen Y Mu S Highly efficient chemoenzymatic synthesis of β1–3-linked galactosides. Chem Commun (Camb) (2010) 46:7507–9. 10.1039/c0cc02850a 20830443 PMC3114946

[151] Nishimoto M Kitaoka M. One-pot enzymatic production of beta-d-galacto pyranosyl-(1 – >3)-2-acetamido-2-deoxy-d-galactose (galacto-N-biose) from sucrose and 2-acetamido-2-deoxy-d-galactose (N-acetylgalactosamine). Carbohydr Res (2009) 344:2573–6. 10.1016/j.carres.2009.09.031 19850285

[152] Armstrong Z Withers SG. Synthesis of glycans and glycopolymers through engineered enzymes. Biopolymers (2013) 99:666–74. 10.1002/bip.22335 23821499

